# Unique Features of Odorant-Binding Proteins of the Parasitoid Wasp *Nasonia vitripennis* Revealed by Genome Annotation and Comparative Analyses

**DOI:** 10.1371/journal.pone.0043034

**Published:** 2012-08-27

**Authors:** Filipe G. Vieira, Sylvain Forêt, Xiaoli He, Julio Rozas, Linda M. Field, Jing-Jiang Zhou

**Affiliations:** 1 Departament de Genètica and Institut de Recerca de la Biodiversitat (IRBio), Universitat de Barcelona, Barcelona, Spain; 2 ARC Centre of Excellence for Coral Reef Studies, James Cook University, Townsville, Queensland, Australia; 3 Evolution, Ecology and Genetics, Research School of Biology, The Australian National University, Canberra, Australian Capital Territory, Australia; 4 Department of Biological Chemistry and Crop Protection, Rothamsted Research, Harpenden, Hertfordshire, United Kingdom; University of Lausanne, Switzerland

## Abstract

Insects are the most diverse group of animals on the planet, comprising over 90% of all metazoan life forms, and have adapted to a wide diversity of ecosystems in nearly all environments. They have evolved highly sensitive chemical senses that are central to their interaction with their environment and to communication between individuals. Understanding the molecular bases of insect olfaction is therefore of great importance from both a basic and applied perspective. Odorant binding proteins (OBPs) are some of most abundant proteins found in insect olfactory organs, where they are the first component of the olfactory transduction cascade, carrying odorant molecules to the olfactory receptors. We carried out a search for OBPs in the genome of the parasitoid wasp *Nasonia vitripennis* and identified 90 sequences encoding putative OBPs. This is the largest OBP family so far reported in insects. We report unique features of the *N. vitripennis* OBPs, including the presence and evolutionary origin of a new subfamily of double-domain OBPs (consisting of two concatenated OBP domains), the loss of conserved cysteine residues and the expression of pseudogenes. This study also demonstrates the extremely dynamic evolution of the insect OBP family: (i) the number of different OBPs can vary greatly between species; (ii) the sequences are highly diverse, sometimes as a result of positive selection pressure with even the canonical cysteines being lost; (iii) new lineage specific domain arrangements can arise, such as the double domain OBP subfamily of wasps and mosquitoes.

## Introduction

Chemical senses are central to the life history of the parasitoid wasp, *Nasonia vitripennis*, commonly known as the jewel wasp. For instance, their courtship behaviour is guided by male [1] and female [2] pheromones and females locate hosts and parasitize the pupae of various fly species using olfactory signals [3], [4]. The wasps also use chemical cues from host pupae to bias the sex ratio of their offspring, manipulate the clutch size and to avoid oviposition on dead hosts, or those containing well-developed parasitoid larvae, pupae or adults [5], [6], [7].

The *Nasonia* genus is mainly comprised of three closely related species of wasps. *N. vitripennis* (Walker) is found throughout the world and is estimated to have diverged from *N. giraulti* and *N. longicornis* approximately 1.0 million years ago (Mya). The three species are normally genetically isolated as the result of *Wolbachia*-induced cytoplasmic incompatibility and laboratory strains, cured of *Wolbachia* are interfertile, provide a useful source of genetic and sequence variation for mapping studies. Consequently, *Nasonia* serves as a useful model system, particularly for the study of the genetics of complex traits and for comparative developmental genetics. The genomes of three *Nasonia* species, *N. vitripennis*, *N. giraulti* and *N. longicornis* have been sequenced [8] and at the time of writing, all other Hymenoptera with a sequenced genome belong to the Aculeata group, which diverged from the Chalcidoida (the clade of parasitic wasps that includes *Nasonia*) approximately 200 Mya ago. The phylogenetic position of *Nasonia* is therefore very important for studies of sequence changes along the apocritan Hymenoptera lineage, which comprises both social and parasitoid groups.

We searched the *Nasonia* genome sequences to gain insight into the evolution and diversity of the odorant binding protein (OBP) family. Insect OBPs are small, water soluble proteins present at a very high concentration (up to 10 mM) in the chemosensillum lymph bathing the dendrites of olfactory nerve cells in antennae [9]. They function as carrier proteins, transporting semiochemicals to olfactory receptors and thus constitute the first molecular recognition step in insect olfaction [9], [10]. Some members of the OBP family have been shown to be involved directly in the olfactory process [11], [12], [13], [14]. However, several studies have reported the expression of OBPs in non-olfactory organs [15], [16], [17], [18] and thus the function of this family of proteins appears to be diverse and context-dependent.

OBPs typically contain six highly conserved cysteine residues, forming disulphide bonds that stabilise the 3-D structures [19], [20], [21], [22], [23]. The presence or absence of these canonical cysteine residues has been used to classify OBPs into three subfamilies: classic OBPs (six canonical cysteines), plus-C OBPs (more than six cysteines) and minus-C OBPs (less than six cysteines) [17], [24], [25], [26], [27]. In *Drosophila melanogaster*, transcripts encoding two OBP dimers (*DmelOBP83cd* and *Dmel83OBPef*) have been identified, with two complete OBP domains each with six conserved cysteines (a total of 12 cysteines). Some OBPs are thought to form homo- and hetero-dimers *in vivo* [28] and thus the fusion of two OBP domains would generate an OBP dimer to form a single gene.

The *Nasonia* genome paper [8] reported a preliminary annotation of 90 OBP-like genes. Here we carry out further analyses of these genes and describe the unique features and the evolutionary origins of the wasp OBPs. A previous study has reported the genome annotation of 90 OBP-like genes in the *N. vitripennis* genome as the supplementary material [8]. Here we carry out further analyses of those OBPs report for the first time the genome annotation and analysis of 90 OBP-like genes in the genome of *N. vitripennis* and describe the unique features and evolutionary origin of the wasp OBPs.

## Results and Discussion

### Annotation of OBPs

**Figure 1:**
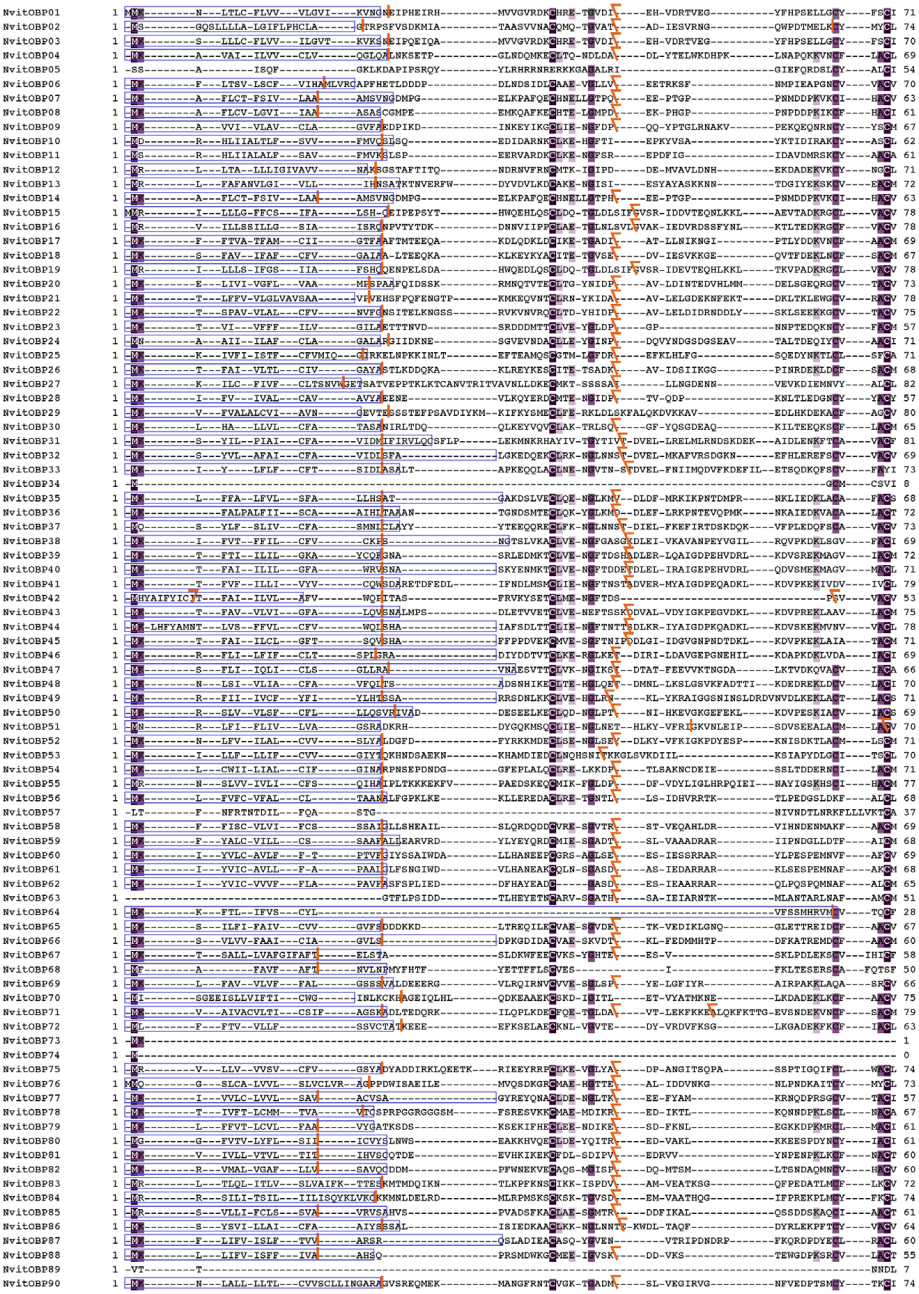
Multiple Sequence Alignment (MSA) of all 90 *Nasonia* OBP sequences. The signal peptides are in skyblue boxes, the conserved residues are highlighted, the characteristic cysteines indicated in purple boxes. The splice sites are labelled with orange separators: vertical ones indicate splice sites between codons; backward slanted separators indicate splice sites within codons after the first base. The double-domain OBPs are NvitOBP38-NvitOBP46 and NvitOBP48.

Genome wide searches of the *N. vitripennis* (Nvit) assembly v1.0 [8] identified 90 sequences [EMBL: HE578186-HE578278] encoding proteins with a high similarity (likely homologous) to insect OBPs which we have named NvitOBPs (Table 1, Figure 1, Figure S3 and S4). Searches against the *N. vitripennis* ESTs and the raw genomic traces did not yield any additional sequences; it is therefore likely that we have identified the full complement of OBP-like sequences in *N. vitripennis*. This is the largest OBP family reported so far in any insect species [29], [30]. By contrast, the honeybee *Apis mellifera* has only 21 OBP genes [31], the fire ant *Solenopsis invicta* only 12 [32] and the pea aphid *Acyrthosiphon pisum* only 15 [33]. As would be expected, based on the very high genomic sequence similarity between the three *Nasonia* species, all 90 sequences identified in *N. vitripennis* have a clear ortholog in *N. giraulti* and *N. longicornis*. Interestingly, we did not find any “plus-C” OBPs [26] in this large OBP repertoire with all *Nasonia* OBPs belonging to the “classic” or the “minus-C” OBP subfamilies [25]. We named *N. vitripennis* OBP genes using the “NvitOBP” prefix followed by a number incremented from the 5′ end of their scaffold to the 3′ end, in increasing scaffold order (Table 1). Of the 90 genes, 59 sequences have full EST support, three are partially supported and the remaining 28 have no EST support, eight of the latter having frameshifts, indicating that these are pseudogenes (Table 1).

**Table 1:** Summary of *N. vitripennis* OBPs. Unless otherwise indicated, the “Status” is protein coding and “EST support” is full.

Name	Scaffold	Status	EST support	Subfamily	Name	Scaffold	Status	EST support	Subfamily
NvitOBP01	1		Yes	Classic	NvitOBP02	1		Yes	Classic
NvitOBP03	1		Yes	Classic	NvitOBP04	3		None	Classic
NvitOBP05	3	pseudogene	None	Classic	NvitOBP06	5		Yes	Classic
NvitOBP07	9		Yes	Classic	NvitOBP08	9		Yes	Classic
NvitOBP09	9		Yes	Classic	NvitOBP10	9		None	Classic
NvitOBP11	9		Yes	Classic	NvitOBP12	9		Yes	Classic
NvitOBP13	9		Yes	Classic	NvitOBP14	9		None	Classic
NvitOBP15	9		Yes	Classic	NvitOBP16	9		None	Classic
NvitOBP17	9		Yes	Classic	NvitOBP18	9		Yes	Classic
NvitOBP19	9		Yes	Classic	NvitOBP20	9		Yes	Classic
NvitOBP21	9		None	Classic	NvitOBP22	9		None	Classic
NvitOBP23	9		Yes	Classic	NvitOBP24	9		None	Classic
NvitOBP25	9		Yes	Classic	NvitOBP26	9		Yes	Classic
NvitOBP27	9		Yes	Minus-C	NvitOBP28	9		Yes	Classic
NvitOBP29	9		None	Classic	NvitOBP30	9		Yes	Classic
NvitOBP31	9		None	Classic	NvitOBP32	9		Yes	Classic
NvitOBP33	9	pseudogene	Yes	Classic	NvitOBP34	9	pseudogene	Yes	Classic
NvitOBP35	9		Yes	Classic	NvitOBP36	9		None	Classic
NvitOBP37	9		Yes	Classic	NvitOBP38	9		Yes	Minus-C
NvitOBP39	9		Partial	DoubleMinus-C(a)	NvitOBP40	9		Yes	DoubleMinus-C(a)
NvitOBP41	9		Yes	DoubleMinus-C(a)	NvitOBP42	9		None	DoubleMinus-C(a)
NvitOBP43	9		None	Double*Minus-C(a)	NvitOBP44	9		Yes	DoubleMinus-C(a)
NvitOBP45	9		Yes	DoubleMinus-C(a)	NvitOBP46	9		Yes	DoubleMinus-C(a)
NvitOBP47	9		Yes	Classic	NvitOBP48	9		Yes	Double
NvitOBP49	9		Yes	Double	NvitOBP50	9		Yes	Classic
NvitOBP51	9		Yes	Classic	NvitOBP52	9		Yes	Classic
NvitOBP53	9		Yes	Classic	NvitOBP54	9		Yes	Classic
NvitOBP55	9		Yes	Classic	NvitOBP56	9		Yes	Minus-C
NvitOBP57	9	pseudogene	None	Classic	NvitOBP58	9		Yes	Minus-C
NvitOBP59	9		Partial	Minus-C	NvitOBP60	9		Yes	Minus-C
NvitOBP61	9		None	Minus-C	NvitOBP62	9		Yes	Minus-C
NvitOBP63	9	incomplete	None	Classic	NvitOBP64	9	incomplete	None	Classic
NvitOBP65	18		Yes	Classic	NvitOBP66	18		Yes	Classic
NvitOBP67	20		Yes	Classic	NvitOBP68	20	pseudogene	None	Classic
NvitOBP69	24		Yes	Classic	NvitOBP70	30		None	Classic
NvitOBP71	30		Yes	Classic	NvitOBP72	30		Partial	Classic
NvitOBP73	30	pseudogene	Yes	Classic	NvitOBP74	33	pseudogene	Yes	Classic
NvitOBP75	40		Yes	Classic	NvitOBP76	126		None	Classic
NvitOBP77	153		None	Classic	NvitOBP78	153		None	Classic
NvitOBP79	153		None	Classic	NvitOBP80	153		None	Classic
NvitOBP81	153		Yes	Classic	NvitOBP82	153		Yes	Classic
NvitOBP83	153		Yes	Classic	NvitOBP84	153		Yes	Classic
NvitOBP85	163		None	Classic	NvitOBP86	174	incomplete	Yes	Classic
NvitOBP87	178		None	Classic	NvitOBP88	178		None	Classic
NvitOBP89	178	pseudogene	None	Classic	NvitOBP90	185		Yes	Classic

* OBP only has some vestiges of its second domain.

(a) On its first domain only.

### Loss of conserved cysteine residues

The highly conserved pattern of cysteine residues in insect OBPs is very important for the disulphide bonding that stabilise the folded OBP structures [19], [20]. Classic OBP structure is composed of 6 cysteine residues forming 3 disulphide bonds, although other forms have also been reported [24], [34]. Interestingly, the wasp OBPs have lost a large number of such conserved cysteines, with a minimum of 22 independent loss events (Table 2). One event involves the loss of three cysteines (C4/C5/C6), 13 events involve the loss of two cysteines (1 for C1/C3; 8 for C2/C5; 1 for C2/C6; 2 for C4/C6; 1 for C5/C6) and eight events involve the loss of a single cysteine (2 for C1; 2 for C2; 2 for C5; 2 for C6). We assessed whether the cysteine losses occurred at random or if they preferentially involved disulphide bond-forming cysteines (Table 2). Considering just the 13 events involving two cysteine replacements (58 OBP genes), 11 of them involved cysteines forming disulphide bonds. The probability that 11 out of 13 would specifically affect the two disulphide bond-forming cysteines is extremely low (*p* = 1.065779e^−6^), suggesting that the replacement of one cysteine (if such event is not deleterious) reduces the selective constraints acting on their former partner.

**Table 2:** Conserved cysteine residue losses in OBPs.

Cysteine	No. of events (No. of affected genes)	Affected Genes (one event per line)
C1	2 (3)	NvitOBP31, NvitOBP64, DmelOBp59a.
C2	2 (2)	AgamOBP38b, AgamOBP42a.
C5	2 (8)	NvitOBP38b, NvitOBP39b, NvitOBP40b, NvitOBP41b, NvitOBP42b, NvitOBP44b, NvitOBP45b, ApisOBP11.
C6	2 (2)	DmelOBP73a, AcerASP4.
C1/C3*	1 (4)	AgamOBP34b, AgamOBP35b, AgamOBP36b, AgamOBP37b.
C2/C5*	8 (50)	NvitOBP27, NvitOBP56, NvitOBP58, NvitOBP59, NvitOBP60, NvitOBP61, NvitOBP62, NvitOBP38a, NvitOBP39a, NvitOBP40a, NvitOBP41a, NvitOBP42a, NvitOBP43, NvitOBP44a, NvitOBP45a, NvitOBP46a,DmelOBP8a, DmelOBP99c, DmelOBP99d,DmelOBP44a,AgamOBP39b,LtesOBP8,AmelOBP14, AmelOBP15, AmelOBP16, AmelOBP17, AmelOBP18, AmelOBP19, AmelOBP20, AmelOBP21, TcasOBP02, TcasOBP03, TcasOBP04, TcasOBP05, TcasOBP06, TcasOBP07, TcasOBP08, TcasOBP09, TcasOBP10, TcasOBP11, TcasOBP12, TcasOBP13, TcasOBP14, TcasOBP15, TcasOBP22, TcasOBP23, TcasOBP24, TcasOBP33, TcasOBP34, TcasOBP44.
C4/C6*	2 (2)	AgamOBP40a,AgamOBP45b.
C2/C6	1 (1)	NvitOBP69.
C5/C6	1 (1)	AgamOBP65.
C4/C5/C6	1 (1)	AgamOBP16.

* Cysteine pairs forming disulphide bonds in the OBPs. Incomplete sequences were excluded.

### Double-domain OBPs

We have identified 10 unusual *N. vitripenis* sequences encoding putative OBPs (OBP38-OBP46 and OBP48) with only little sequence similarity with the OBPs of other insect species. However, these 10 OBPs have significant similarity with those of the other wasp OBPs, which are in turn related to the classical OBPs of other insects. Moreover, the 10 *N. vitripenis* genes are found in the middle of a genomic cluster located on scaffold 9 containing predominantly OBP genes (Table 1). Nine of these non-standard OBPs (all except OBP43) are exceptionally long (more than 250 amino acids) and are formed by two OBP domains arranged in tandem. One of the two domains, however, has undergone extensive loss of the canonical cysteines and become a “minus-C OBP”. These double-domain OBPs are thus different from the dimer OBPs of *D. melanogaster* (Dmel), such as DmelOBP83cd and DmelOBP83ef, which have two complete OBP domains with no cysteine losses. This domain organization fits well with the functional hypothesis of OBP dimerization [28], [35], [36]. Moreover, a number of ESTs support the fact that the transcripts of these genes contain both OBP domains.

### Domain definition

**Figure 2:**
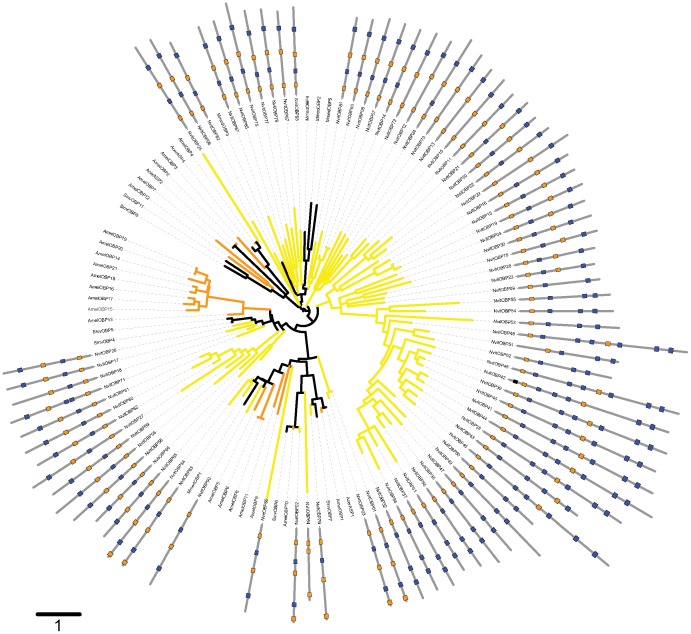
Mid-point rooted phylogenetic relationships of *Nasonia vitripennis* (light brown) and *Apis mellifera* (orange) OBPs. The outer ring shows the intron/exon structure in the coding region (intron phases are represented by colour-coded crossed bars: dark orange, phase 0; blue, phase 1; black, phase 2. The scale bar represents 1 amino acid substitution per site. The tree is displayed using the iTOL webserver (Letunic and Bork 2007). The accession numbers of OBPs used are listed in Table S1.

**Figure 3:**
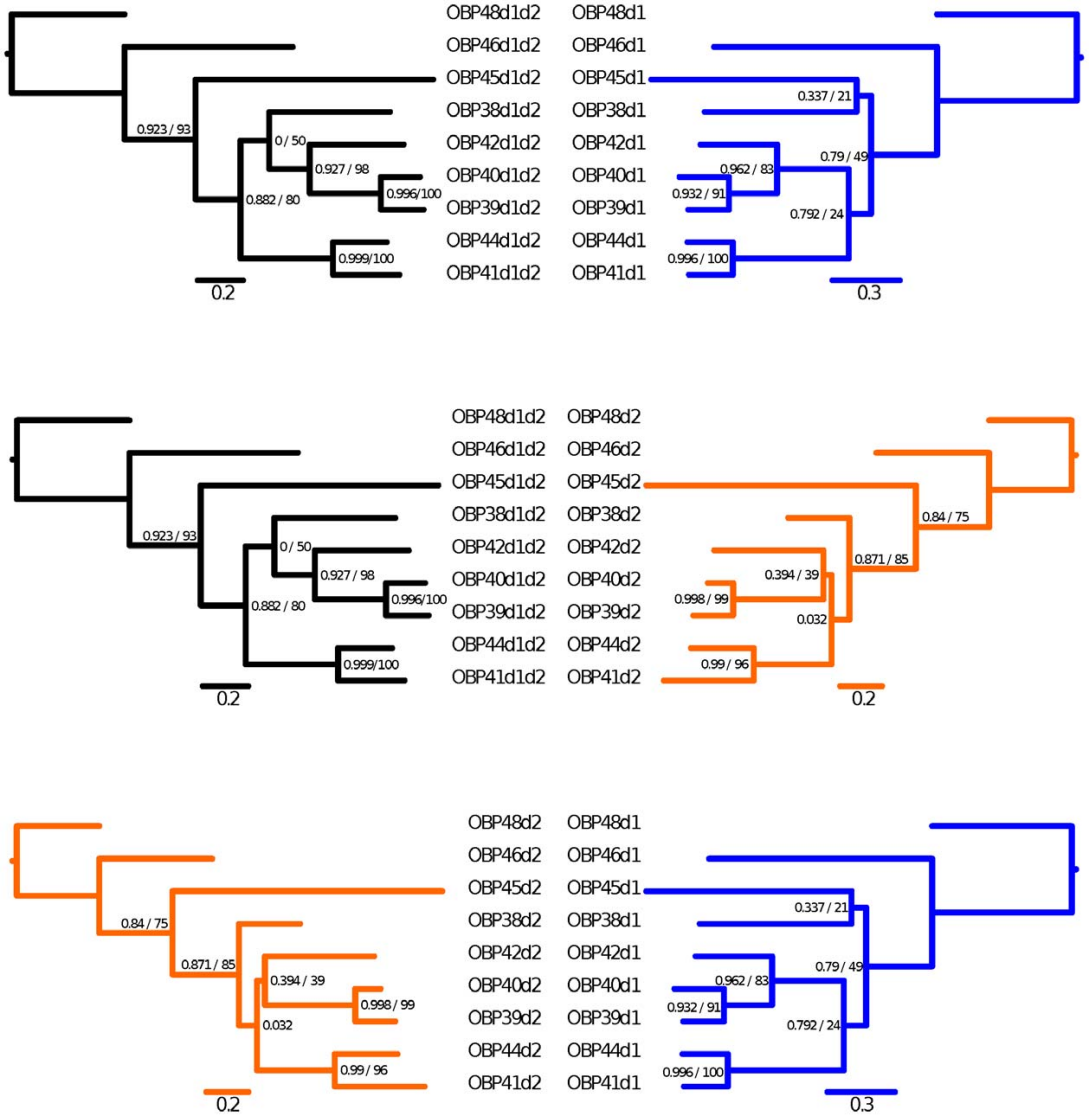
Comparison of the phylogenetic tree topologies of double-domain OBPs. The phylogenetic trees built using the full-length Nasonia double-domain OBPs are depicted in black, and those with information of the first domain or second domain in dark blue or dark orange, respectively. A) Full-length OBPs (left) compared with the first OBP domain (right). B) Full-length OBPs compared with the second OBP domain. C) The second domain compared with the first domain. Branch support values represent Bayesian and bootstrap, respectively. Scale bars represent amino acid substitutions per site.

We defined the two domains of the double-domain wasp OBPs using the splicing pattern, sequence similarity and cysteine profile (Figure 1). In fact, the two domains have, in almost all cases, the same gene structure, which is similar to the closely-related classic OBPs (except for the signal peptides) (Figure 2 and 3). Excluding the signal peptides, we define the 1^st^ domain from positions 25 to 186 and the 2^nd^ domain from 187 to ∼240 of the multiple sequence alignment (Figure 1).

**Figure 4:**
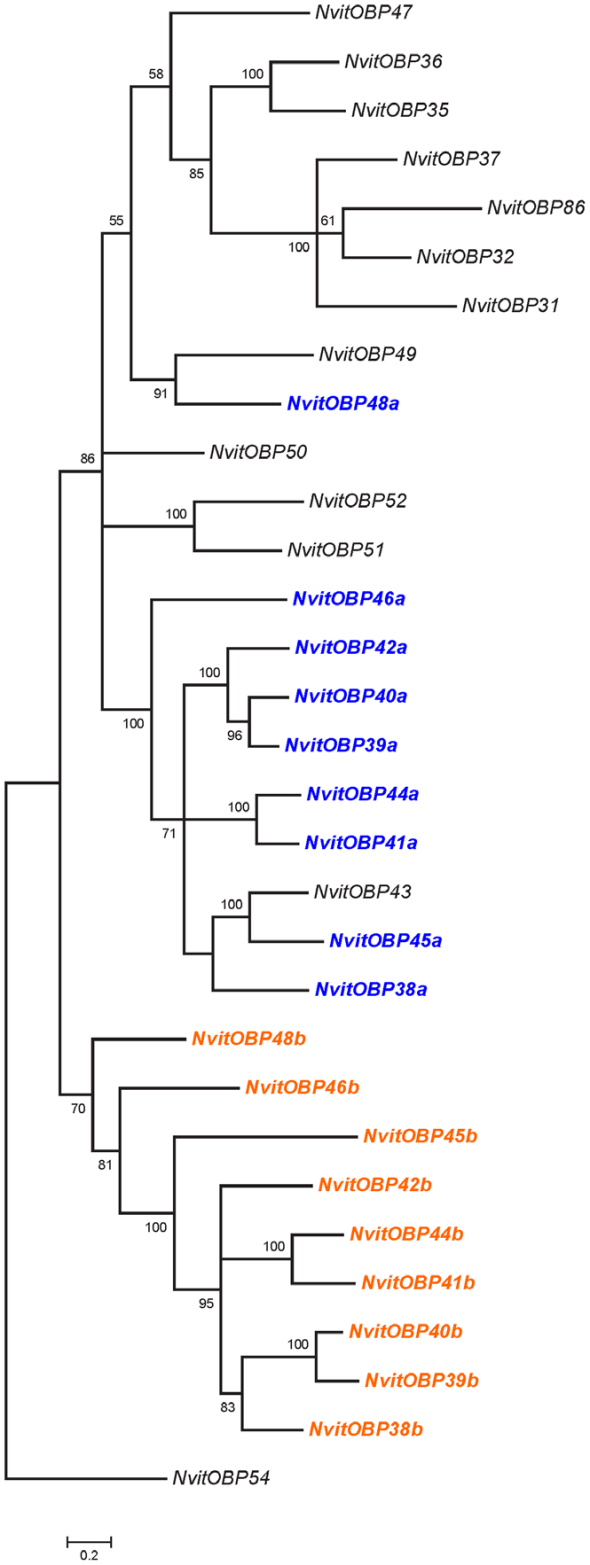
Phylogenetic relationships of *Nasonia*'s double-domain OBPs with their closest Classic OBPs. Double-domain OBPs were split in the two encompassing domains (domain 1 in dark blue; domain 2 in dark orange).

Interestingly, the gene cluster containing the double-domain OBPs also includes two single-domain proteins (NvitOBP43 and NvitOBP47) that appear to have different evolutionary histories. The phylogenetic analysis suggests that NvitOBP47 is a classic OBP, with just one domain, whilst the NvitOBP43 sequence clusters with the 1^st^ domain of other double-domain OBPs (Figure 3 and Figure 4). Furthermore, the NvitOBP43 gene has several features reminiscent of the 2^nd^ domain i.e. the 1^st^ exon, the splice site and the 1^st^ cysteine. It is therefore likely that NvitOBP43 is a former double-domain OBP that has lost most of its 2^nd^ domain.

### Overlapping transcripts and expressed pseudogenes

**Figure 5:**
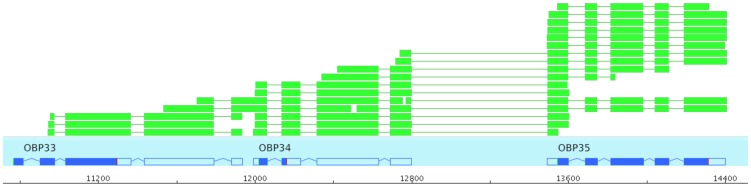
Overlapping transcripts. In green: *Nasonia vitripenis* ESTs, in blue: gene models predicted based on these ESTs.

An analysis of all publicly available *Nasonia* ESTs revealed a region on Scaffold 9 (between positions 2,440,886 and 2,447,816), within the double domain OBP gene cluster, containing several ESTs that partially overlap two pseudogenes (*NvitOBP33* and *NvitOBP34*) and one gene (*NvitOBP35*). Two of these ESTs span across *NvitOBP33* and *NvitOBP34*, and 11 ESTs cover exons of both *NvitOBP34* and *NvitOBP35* (Figure 5). *NvitOBP33* and *NvitOBP34* are predicted to be pseudogenes, and this prediction is supported by ESTs. It is possible, however, to construct alternative genes models that do not contain the frame-shift and premature stop codons found in these genes (Figure S1), but still contain only canonical intron-exon junctions. We used primers designed to test for the presence of transcripts from these genes but none were detected (Figure S2).

### Evolutionary origin of the double domain OBPs

**Figure 6:**
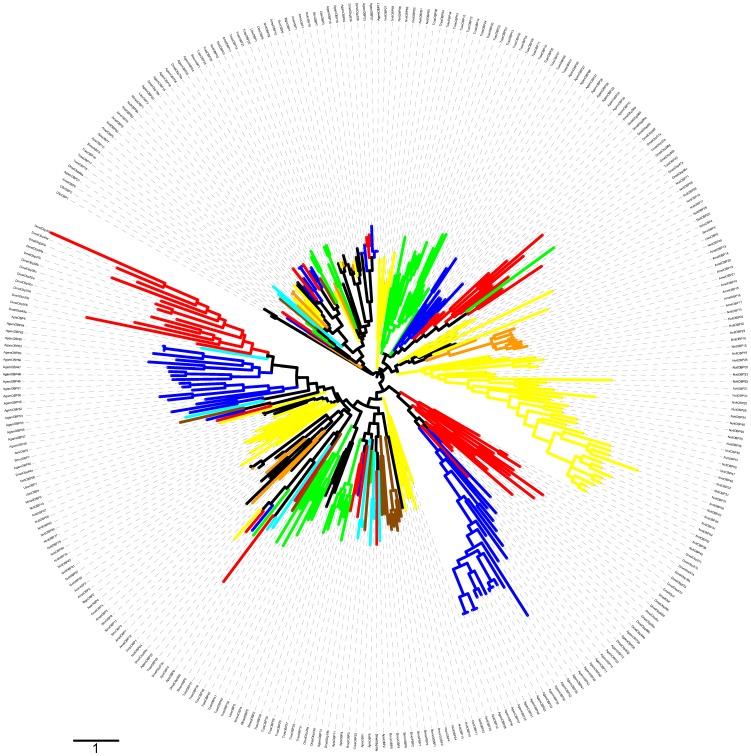
Phylogenetic relationships of OBPs from some insect species. The mid-point rooted tree includes OBP sequences from *Drosophila melanogaster* and *Drosophila mojavensis* (Dmel, Dmoj; red branches), *Anopheles gambiae* (Agam; blue branches), *Bombyx mori* (Bmor; brown branches), *Tribolium castaneum* (Tcas; green branches), *Apis mellifera* (Amel; orange branches), *Nasonia vitripennis* (Nvit; yellow branches), *Pediculus humanus* (Phum; pink branches) and *Acyrthosyphon pisum* (Apis; cyan branches). The scale bar represents 1 amino acid substitution per site. The image was created using the iTOL web server (Letunic and Bork 2007). The accession numbers of the OBPs are listed in Table S1.

Phylogenetic analyses revealed that all double-domain *Nasonia* OBPs are monophyletic, suggesting a unique origin of these proteins. Indeed, each domain is also monophyletic and the topologies of the trees based on the separate domains are consistent with the trees constructed with the full length sequences (Figure 3 and Figure 4). There are two possible mechanisms for such double-domain OBPs, either internal gene duplication or gene fusion between two physically close paralogs. The existence of overlapping transcripts in the cluster containing these genes (see previous section) might suggest that the latter mechanism is more likely. Also, the *Nasonia* double-domain OBPs clearly cluster together with some classic OBPs with a reasonable SH (Shimodaira–Hasegawa likelihood ratio test [37]) support (Figure 2), suggesting that double-domain OBPs evolved from a single event that merged two closely related genes. Moreover, all the *Nasonia* double-domain OBPs are located in a cluster located on scaffold 9 (Table 1), suggesting that this expansion occurred after the merging of the two domains. Remarkably, the mosquitoes *Anopheles gambiae* and *Aedes aegypti* also have a cluster of double-domain OBPs (the atypical OBPs) [38], [39] and their evolutionary history is broadly similar to what we have seen in *N. vitripennis*, with a putative origin from a single duplication/fusion event, as shown on Figure 6.

The main lineage-specific expansion of the OBP family in *Nasonia* includes the double-domain genes (Figure 2). We carried out an analysis of the selective selection pressure on these genes using the codeml software of the PAML package. As indicated in Table 3, this showed that the M2s model (with positive selection) has a higher likelihood than the M1a (purifying selection) but the difference is not statistically significant (p = 0.7). The M8 model, however, has a significantly higher likelihood than both the M7 (purifying) (p = 0.004) and M8a (purifying and neutral) models (p = 0.02), but none of the sites reach the 95% confidence level (BEB analysis). This suggests that positive selection pressure has probably played a role in the diversification of the sequences in this lineage specific expansion, but such inference based on such diverged protein sequences is to be taken with caution [40].

**Table 3:** Log-likelihood and statistical significance of various models of selection pressure on the sequence of the *Nasonia* double domain OBPs, obtained with the codeml software of the PAML package.

Models	Log likelihood (lnL)	P-values
M1a	−7077.78	
M2a	−7077.07	(M1a vs M2a)−2lnL = 0.71, df = 2, p = 0.7
M7	−7057.53	
M8	−7051.00	(M7, M8)−2lnL = 11.07, df = 2, p = 0.004
M8a	−7055.78	(M8a, M8)−2lnL = 7.58, p = 0.02

### Relationships with other insect OBPs

Phylogenetic trees for OBPs from Hymenopteran insects (Figure 2) and from other insects with genome or genomic sequences publically accessible were constructed using Bayesian phylogenetic inference (MrBayes v3.2) (Figure 6). We considered that the inference procedure had converged when the average standard deviation of split frequencies was smaller than 0.05 and 0.01 for ‘all insect’ and ‘hymenoptera’ trees, respectively. The trees show a number of highly supported terminal relationships between genes in tandem and closely related genes and several lineage specific expansions, both hinting at extensive expansion after speciation by tandem gene duplication.

We have found three orthologs of *Nasonia* OBPs in *A. mellifera (NvitOBP76-AmelOBP1, NvitOBP02-AmelOBP10, NvitOBP90-AmelOBP5)* (Figure 2) and two orthologous groups with members in several of the insects studied (*DmelObp59a-AgamOBP29-TcasOBP45-NvitOBP64* and *DmelObp73a-ApisOBP4*) (Figure 6). This high conservation across insect species suggests that these OBPs have an important function for insects.

## Conclusions

Using the OBP sequence motif search [23], [26], [33], [39], homology searching [25], [41], [38], [42] and genomic sequence analyses [35], [30] we have annotated 90 genes encoding putative OBPs in *N. vitripennis*. This is the largest OBP gene family so far reported in insects and has allowed us to identify some of unique features and to be able to study evolution origin of these features. This exceptionally high number of OBPs mirrors the large number of genes encoding olfactory receptors in this species (over 200 ORs) [8]. It has been proposed that the expansion of these gene families related to olfaction has an origin in the need to detect and discriminate between a number of diverse odours for food and reproduction and to avoid dangers [43]. In the absence of functional evidence, however, it is unclear how many of the *Nasonia* OBPs are truly involved in olfaction, and whether the OBP expansion in *Nasonia* is necessarily related to any olfactory role. Further experiments are needed to determine which *Nasonia* OBPs are expressed in olfactory tissues, and what type of molecule they carry.

All *Nasonia* OBPs belong to the “classic” subfamily with no “Plus-C” OBPs but a large number which have lost at least one of the characteristic six conserved cysteines. Our most striking finding is a group of double-domain OBPs with little sequence similarity to OBPs of other species but with substantial similarity with the other NvitOBPs. These double-domain OBPs are thus different from the fusion of two classic OBP domains in that one of the two domains has undergone extensive loss of the canonical cysteines and become equivalent to “minus-C OBPs”. This is supported by the finding that the two domains of these NvitOBPs have the same gene structure, similar to the closely related classic OBPs, and are monophyletic. The results suggest that double-domain OBPs evolved from a single event. It is possible these functional double-domain OBP proteins may have acquired an equivalent function to that performed by two ‘classic’ OBPs acting as dimmers.

Taken together, the multiple occurrences of canonical cysteine losses, and the independent emergence of double domain OBPs in wasps, flies and mosquitos, represent a striking case of convergent evolution of protein structures. The main challenge remains to understand the functional significance of these structural changes.

## Materials and Methods

### OBP identification and annotation

A list of all described OBP-like protein sequences was built as described in Forêt and Maleszka (2006). The sequences were then used to build models for PSI-BLAST [44] and HMMER [45] to search the *N. vitripennis* genomic scaffolds [8]. The PSI-BLAST and HMMER models were iteratively updated with each newly identified sequence. Gene models were constructed and manually curated using the Apollo genome annotation software [46].

### Nucleic acids analyses

Whole adult *N. vitripennis* were homogenised in 250 µL genomic DNA extraction buffer (100 mM Tris-HCl pH 9.0, 100 mM EDTA, 1% SDS) in 1.5-mL eppendof tubes. The extraction mixture was heated at 70°C for 30 min, mixed with 35 µL of 8 M KAc solution, incubated on ice for 30 min. The supernatant containing DNA was obtained by centrifugation at 13,000×g for 10 min, and then extracted further with 280 µL chloroform∶phenol 1∶1. The DNA sample was treated with 2 µL RNase (10 mg/mL) at 37°C for 15 min, extracted again with 250 µL chloroform, and finally DNA was precipitated with 2.5 volume of 100% ethanol.

RNA from whole *N. vitripennis* adults was extracted using Trizol reagent (Invitrogen) according to the manufacturer's protocol. Reverse transcriptions were carried out using SuperScript II (Invitrogen). The cDNA for RT-PCR was prepared with an oligo(dT)_15_ primers (Promega) and SuperScript™ II Reverse Transcriptase RNAase H^−^ (Invitrogen Life Technologies), and used as the PCR template.

The PCR amplifications contained 14.5 µl sterile water, 2 µl (10×) PCR buffer including 15 mmol/L Mg^2+^, 0.4 µl dNTP mixture (10 mmol/L), 0.5 µl each of forward and reverse primers (25 mmol/L), and 0.1 µl HotStarTaq® DNA polymerase (5 U/µl, Qiagen, Chatsworth, CA) and were done in a Hybaid thermocycler with thermo-cycling program: 15 min at 95°C followed by 35 cycles of 40 sec at 94°C, 40 sec at 53°C, and 40 sec at 72°C, with 7 min at 72°C after the last cycle. The PCR primers are Primer_F1 (5′-CCCAGGATAAGCAGTTTAGCTG-′3) on the second exon of the 3′-end of *NvitOBP33*, Primer_R2 (3′-TCATTTTCGACGAGCTGCTG-5′) on the first exon of the 5′-end of *NvitOBP34* and Primer_R1 (3′-ATTTAGAGGATTACTGCGACGC-5′) on the last exon of *NvitOBP35*.

### Phylogenetic analysis

Amino acid sequences were aligned with MAFFT v6 [47], removing positions with over 95% gaps. Phylogenies were inferred using maximum likelihood (ML; with PhyML v2.0, [48]) and Bayesian inference using MrBayes v1.2 [49]. For these analyses the LG amino acid substitution matrix was used and positions with over 95% gaps were removed, as preliminary MrBayes runs rapidly converged to this model. Support for maximum likelihood phylogenies were assessed with the likelihood-based SH test [37] using 100 bootstrap replicates. Each Bayesian analysis was run for 10,000,000 iterations, using two parallel runs with four chains each. The first 2,500,000 iterations were discarded as “burnin”. All analyses reached convergence, as indicated by the average standard deviation of split frequencies being smaller than 0.05 or 0.01 for the ‘all insect’ and the ‘Hymenoptera’ trees, respectively. The trees were displayed with iTOL [50] and FigTree (http://tree.bio.ed.ac.uk/software/figtree/). Analyses of selective constraints were conducted using the *codeml* program from the PAML v4 software package [51].

### Conserved cysteine profiles

We inferred cysteine loss events by minimizing the number of events given the OBP phylogenetic gene tree (a maximum parsimony criteria). We tested if the cysteine loss events had occurred at random or had preferentially involved disulphide bond-forming cysteines. Assuming that each OBP has 6 cysteines, forming three disulphide bonds, after losing the first cysteine the probability of a second loss in a ‘bond’ or ‘non-bond’ cysteine is q = 1/5 and p = 4/5, respectively. We can thus use a binomial distribution to compute the probability that given a number of X events involving two cysteine replacements Y (or more) involve cysteines forming disulphide bonds by the binomial distribution.

### Defining OBP domains

We delimited the OBP domains using sequence similarity, splicing pattern and cysteine profile information. We first determined the approximate boundary of the two domains by a dotplot analysis (using the web-based Dotlet program; [52]) between several single-domain and double-domain proteins. To pinpoint the most probable boundary between the domains we complemented the dotplot analysis with the splice site distribution and cysteine profiles.

## Supporting Information

Figure S1 **Alternative models for NvitOBP33 and NvitOBP34.**
(DOCX)

Figure S2 **PCR products of the overlapping transcript within a genomic region containing three OBP genes from the cDNA (Lane 1 and 3) and gDNA (Lane 2 and 4) with the primer pair crossing whole region containing three OBP genes (Lane 1 and 2), and with the primer pair crossing the region containing** ***NvitOBP33*** **and** ***NvitOBP34*** **(Lane 3 and 4).**
(DOCX)

Figure S3 **The derived protein sequences of all 90** ***Nasonia vitripennis*** **OBPs.**
(PDF)

Figure S4 **The Coding DNA sequences and EMBL entries of all 90** ***Nasonia vitripennis*** **OBPs.**
(DOCX)

Table S1 **Accession numbers of protein sequences cited in the manuscript.**
(PDF)
