# Supplementary figures and images for: Unique Features of Odorant-Binding Proteins of the Parasitoid Wasp Nasonia vitripennis Revealed by Genome Annotation and Comparative Analyses
Source: PLoS One. 2012 Aug 27;7(8):e43034. doi: 10.1371/journal.pone.0043034 (PMC3428353; doi:10.1371/journal.pone.0043034)

**Supplementary Figure S1.**

**
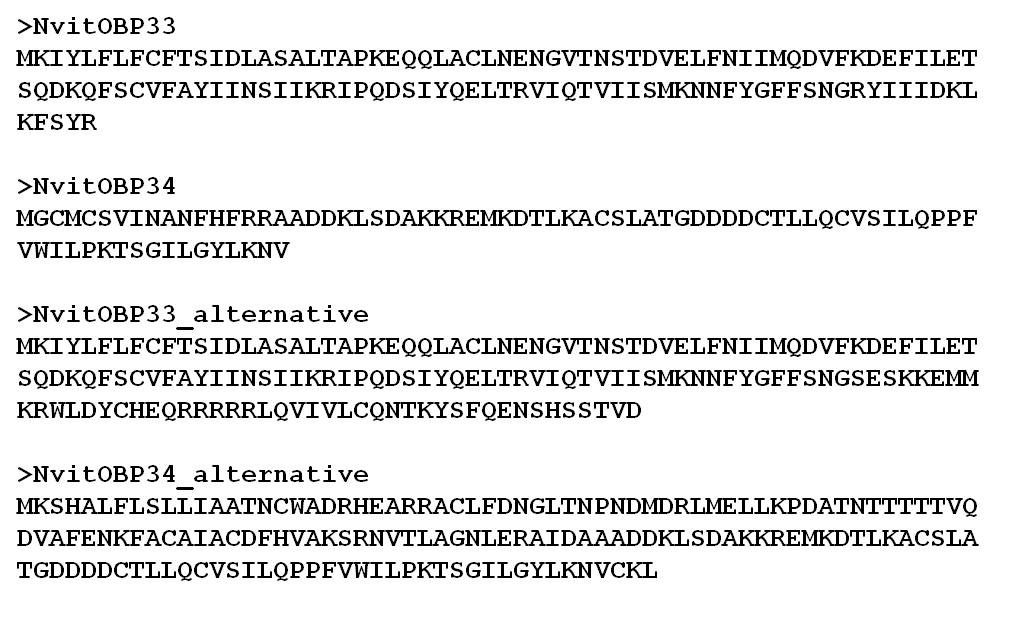
**

Supplement: Figure S1 — Alternative models for NvitOBP33 and NvitOBP34. (DOCX) [file pone.0043034.s001.docx]
