# Supplementary material for: Unique Features of Odorant-Binding Proteins of the Parasitoid Wasp Nasonia vitripennis Revealed by Genome Annotation and Comparative Analyses
Source: PLoS One. 2012 Aug 27;7(8):e43034. doi: 10.1371/journal.pone.0043034 (PMC3428353; doi:10.1371/journal.pone.0043034)
Supplement: Figure S2 — PCR products of the overlapping transcript within a genomic region containing three OBP genes from the cDNA (Lane 1 and 3) and gDNA (Lane 2 and 4) with the primer pair crossing whole region containing three OBP genes (Lane 1 and 2), and with the primer pair crossing the region containing NvitOBP33 and NvitOBP34 (Lane 3 and 4). (DOCX) [file pone.0043034.s002.docx]

**Supplementary Figure S2.** PCR products of the overlapping transcript within a genomic region containing three OBP genes from the cDNA (Lane 1 and 3) and gDNA (Lane 2 and 4) with the primer pair crossing whole region containing three OBP genes (Lane 1 and 2), and with the primer pair crossing the region containing *NvitOBP33* and *NvitOBP34* (Lane 3 and 4).


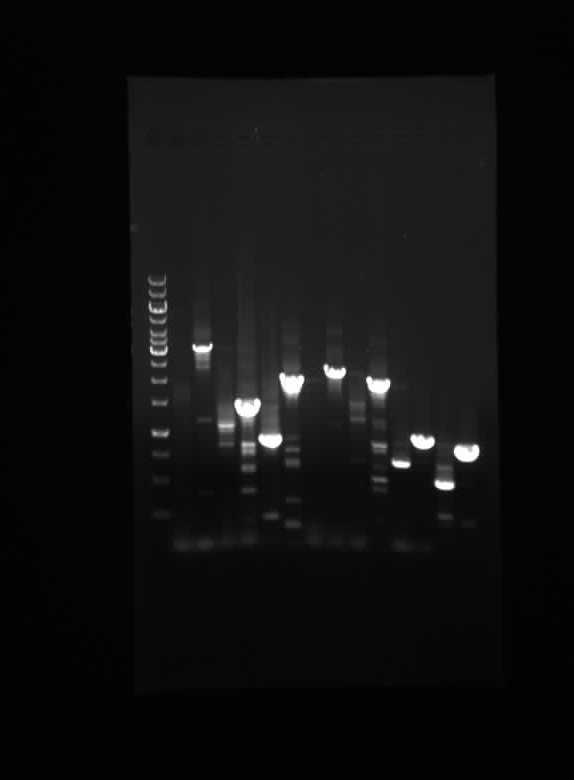


2.5k

250

500

750

1k

1.5k

2k

3k

**1 2 3 4**
