# Supplementary material for: Unique Features of Odorant-Binding Proteins of the Parasitoid Wasp Nasonia vitripennis Revealed by Genome Annotation and Comparative Analyses
Source: PLoS One. 2012 Aug 27;7(8):e43034. doi: 10.1371/journal.pone.0043034 (PMC3428353; doi:10.1371/journal.pone.0043034)
Supplement: Figure S3 — The derived protein sequences of all 90 Nasonia vitripennis OBPs. (PDF) [file pone.0043034.s003.pdf]

NvitOBP01

MMKNLTLCFLVVVLGVIKVNGNEIPHEIRHMVVGVRDKCHRETGVDIEHVDRTVEGYFHPSELLGCYFSC
IFNHFDLLDKDGHLDWDKLVPRIPESFKEHADEMIAACRSTTGKDPCDSALNIVQCFQKTNPSKYFVI

NvitOBP02

MSGQSLLLLALGIFLPHCLAGTRPSFVSDKMIATAASVVNACQMQTGVATADIESVRNGQWPDTMELK
CYMYCLWEQFGLIDEKRELSLNGMLTFFQRIPAYRVEVEKAINECKALATGDTCEYAYTFNKCYAERSPRTYYLF

NvitOBP03

MKSLLLCFLVVILGVTKVKSNEIPQEIQAMVVGVRDKCHRETGVDIEHVDRTVEGYFHPSELLGCYFSCIFN
HFNLLDNDGHLDWVKVVNVIPPSFKDHADEMIAACKTTTGKDPCDSAVNIVQCFQKTNPAKYFVI

NvitOBP04

MKAVAIILVVCLVQGLQALNKSETPGLNDQMKECLTQNDLDADLYTELWKDHPKLNAPQKKVNCFLACL
YKKVGALSADGAIVLPEGLIEERIINWSPELREKCKKQAGDDVCELAGCLDKPNGFLSATV

NvitOBP05

SSAISQFGKLKDAPIPSRQYYLRHRRNRERKKGAGALRIGIEFQRDSLCYALCIAFLAADIDPTAEIEIDRSGS
RAMRWIVDEDFPRERHRPTANCVTRTHNAITSCLGSFPLVRCT

NvitOBP06

MKFLTSVLSCFVIHAMLVRCAPFHETLDDDPDLNDSIDLCAAEVGLLVEETRKSFNMPIEAPGNCVVACV
WKKIGLMELDGKIVKEEMISSLHPTLKQMPNITPIHEDDFYHCVDEANDYEGGCIVVSEYFKCIIRDLFNQLSI

NvitOBP07

MKAFLCTFSIVLAAAMSVNGDMPGELKPAFQECHNELLGTPQEEPTGPPNMDDPKVKCIHACVAKKIG
HMVDGKIVAEKEIESAKQHMPNADNSLTDKITECANKANEQSDECEVSAAFHKCIVEKVGPPEHHH

NvitOBP08

MKAFLCVLGVIIAAASASCGMPEEMKQAFKECHTELGMPDEKPHGPPNPDDPKIKCFHACIMKKAGKM
VDGKLDADKEIEFAKKRMPNADDSMIEKITECVKTANEQSDECEVAGAMHKCIMEKVGSPPHHHRH

NvitOBP09

MKAVVIVLAVCLAGVFAEDPIKDINKEYIKGCLIENGFDPQQYPTGLRNAKVPEKQEQNRNCYYSCMMK
KMNLMKADGSLNEDALRQKFNMNLDTLGKALSTCKDQVKDDKCKLAACLMANRGA

NvitOBP10

MDRHLIIALTLFSVVFMVQSLSQEDIDARNKCLKEHGFTIEPKYVSAYKTIDIRAKCYASCLMRETGVVKED
GSIDLNKVLEKISDSENKTLDEVVKKSFIPCTEKKGDNDCDTGHQILTCIVATISILKESMKIV

NvitOBP11

MSRHLIIALALFSAVFMVKSLSPEERVARDKCLKENGFSREPDFIGIDAVDMRSKCYAACALRGYGIMKED
SSIDINKILEHISDTKNKDIDVKKSLIIPCAEKKGETDCDTGYLITNCVALAVRKLDK

NvitOBP12

MRLLTALLLIGIVAVVNAKSGSTAFTITQNDRNVFRNCMTKIGIPDDEMVAVLDNHEKDADEKVKCYNGC
LYKAFKVIKDDGTVDTEAAIKFFKVEDMESDKNIIVKCSNESNSNKEKNDCDTAQTMESCYYKLKKEQ

NvitOBP13

MRLFAFANVLGIVLLIHNSATKTNVERFWDYVDVLKDCAKENGISIESYAYASKKNNTDGIYEKSKCVEAC MFKSHKIMRPDGTIDMEKAIEHLLTGNPGEKRDLMKKNIESCEIPNGDNECEVAHTMVKCALGYD

NvitOBP14

MKAFLCTFSIVLAAAMSVNGDMPGELKPAFQECHNELLGTPHEEPTGPPNMDDPKVKCIHACVAKKIG HMVDGKIVAEKEIESAKQHMPNADNSLTDKITECANKANEQSDECEVSAAFHKCIVEKVGPPEHHH

NvitOBP15

MMRILLLGFFCSIFALSHQEIPEPSYTHWQEHLQSCLDQTGLDLSIFGVSRIDDVTEQNLKKLAEVTADKRG CLVACVFQKQGMISKEGVLQANPPRPDPTTKLETTFEEAIAACRSEKNFCKLGNCLFEIYFKYK

NvitOBP16

MRVILLSSILLGSIAISRQNPVTYTDKDNNVIIPPCLAETGLNLSVLGVAKIEDVRDSSFYNLKTLTEDKRGCF VACVYKKLGIITEENVLINDRVIPPGVAVPKKKLATAFEDATEACRAQKDLCKLGNCLYEIYFF

NvitOBP17

MKFFTVATFAMCIIGTFAAFTMTEEQAKDLQDKLDCIKETGADIATLLNIKNGIPTLYDDKVNCFAACMLE KFNIMKPDGSMDETVARLRASKSMSQEKVDRVLSSCKSEVGKDKCETGGKILECLMKNDAVPILS

NvitOBP18

MKSFAVIFAFCFVGAIAALTEEQKAKLKEYKYACITETGVSEDVIESVKKGEQVTFDEKLNCFSACMLKKVGI MNADGTVNEEVARAKVPQDLPKDKVDQVINTCKAEVGKDSCETGGKVLACLMKTKAVSVLH

NvitOBP19

MRILLLSIFGSIIAFSHQQENPELSDAHWQEDLQSCLDQTGLDLSIFGVSRIDEVTEQHLKKLTKVPADKRG CLVACVFQKQGMISKEGVLQNNPPHPDPTTKFETTFEDAIAVCRAEENFCKLGNCLFGIYFNYEI

NvitOBP20

MKELIVIVGFLVAAMPSPAAFQIDSSKRMNQTVTECLTGYNIDPAVLDINTEDVHLMMDELSGEQRGCV TACVYKGFDWLKDDGSLDIDALTMDEDPEDTAEFIKDIVDCRNKVGTEACKFFHCLDTKGT

NvitOBP21

MKTLFFVVLGLVAVSAAVPVEHSFPQFENGTPKMKEQVNTCLRNYKIDAAVLELGDEKNFEKTDKLTKLE WGCVRACVYKGANFMRADGSLDIEVLTDGDEPEDKKKFESVVGICRAEAGKDDCKFFQCMDEKDDS

NvitOBP22

MKTSPAVVLALCFVNVFGNSITELKNGSSRVKVNVRQCLTDYHIDPAVLELDIDRNDDLYSKLSEEKKGCV TACVYRGFNWLRPDGSLDIDLLCEGETPEESEAERKRYTKIVAECRAEVGKDDCKFFNCLNLKDL

NvitOBP23

MKTVIVFFFILVGILAETTTNVDSRDDDMTTCLVEYGLDPGPNNPTEDQKNCYFACMFKTIGYMKKDGSF NLDLILSDAYRSEKRVESKRKLDNIVSMCKQRAGNDICKLAGCYQEHRN

NvitOBP24

MNAAIIILAFCLAGALARGIIDKNESGVEVNDACLLEYGINPDQVYNDGSDGSEAVTALTDEQIYCVAACIY KDYGIMRPNGTIDTEKADSYFGEDDSRERDIFFAVYNACSEGRVGCKLVQCMFSELKNHWGSSTSDSKKELKPLFN RPDFIRAN

NvitOBP25

MKKIVFIISTFCFVMIQGIRKELNPKKINLTEFTEAMQSCGTMLGFDREFKLHLFGSQEDYNKTLCLSFCALRKLKFYVIEDEIKKELAHVRNARLKEEVYKALDVCKHLLDDPCKLFDCFFDYAKVVDAENESLNLSKFFSEGSL

NvitOBP26

MKTFAIVLTLCIVGAYASTLKDDQKAKLREYKESCITETSADKAVIDSIIKGGPINRDEKLDCFSACMLKKIGIMRPDGSIDVESARAKAATTNVDVAKANEVIDKCKDLKGKDTCETGGAVFGCFITNKDFPVLN

NvitOBP27

MKKILCFIVFCLTSNVWGETSATVEPPTKLKTCANVTRITVAVNLLDKECMKTSSSSAILLNGDENNVEVKDIEMNVYALCLLQKSSIMNEQGKINLNFDIFKIVKNLYKRTDQRGFGLAFIIKSLEKCRQTDGPDQFSTATKIMKCLLDNQKTVIRCDQH

NvitOBP28

MKIFVIVALCAVAVYAEENEVLKQYERDCMTENGIDPTVQDPKNLTLEDGNCYYACYFKKFGIIKEDGSYDVAAIKEKYSKPNSVEAVQKKLDEITQTYCQDKVGNHCNLAACLSKISKEQWKI

NvitOBP29

MKVFVALALCVIAVNGEVTESSSTEFPSAVDIYKMKIFKYSMECLFERKLDLSKFALQKDVKKAVEDLHKDEKACFAGCVFKKLGAMNDDGTFNEDKLFMGATAETLPIFKQTHDAAVKHCTDKVGKDELCKFAACIVIQAPAYASSLNATSGI

NvitOBP30

MKLHALLVLCFATASANIRLTDQQLKEYVQVCLAKTRLSQGFYQSGDEAQKILTEEQKSCFLACMFKRTGIIDHDGSVNLKLGDEELPRTPAIEACITTAKEDICKLAICLHKTGKFSITSVADSPRYPRYH

NvitOBP31

MKSYILPIAICFAVIDMIFIRVLQCSFLPLEKMNKRHAYIVTGYTIVTDVELLRELMLRNDSKDEKAIDLENKFTCAVACFSDAKINVSREEIKSDLMNTLDTCHQKDEGDNCNLLECVKVLIPPFKALLIFA

NvitOBP32

MKSYVLAFAICFAVIDLSFALGKEDQEKCLRKNGLNNSTDVELMKAFVRSDGKNEFHLEREFSCVVACVIDERRTEDNVNTSTYQLLTDLISEAHNKIPDEQWRDMKTTLDKCHQQDEGDDCKLLYCVKILRDPFKELIVSFD

NvitOBP33

MKIYLFLFCFTSIDLASALTAPKEQQLACLNENGVTNSTDVELFNIIMQDVFKDEFILETSQDKQFSCVFAYIINSIIKRIPQDSIYQELTRVIQTVIISMKNNFYGFFSNGRYIIIDKLKFSYR

NvitOBP34

MGCMCSVINANFHFRRAADDKLSDAKKREMKDTLKACSLATGDDDDCTLLQCVSILQPPFVWILPKTSGILGYLKNV

NvitOBP35

MKLFFALFVLSFALLHSATGAKDSLVECLQENGLKMVDLDFMRKIKPNTDMPRNKLIEDKLACAFACSFNRDNSWKDENVFTFMTDVIKKDYRIPVGLKKQMLDTLKSCNAEAKGDDCTLLQCIKVTRYPFMDFVFLHNTEMKPEYESSQDKQI

NvitOBP36

MKFALPALFIISCAAIHLTAAANTGNDSMTECLQKYGLKMDDLEFLRKPNTEVQPMKNKAIEDKVACALA CTFKKESQYRSIFPFLKNVLRIDRQIPVNLKKDMLDTLDDCNGEAKGNDCKLLQCIKITRNPFMNLVFSYGA

NvitOBP37

MQSYLFSLIVCFASMNLCLAYYYTEEQQRECLFKNGLNNSTDIELFKEFIRTDSKDQKVFPLEDQFSCAVAC VFNLGKPDPSEDRIYHKLLYSIKNDDQIPGELKRYMMDKLDHCHRQDEGDDCKLFICIKLFRPPFKETIISIE

NvitOBP38

MKIFVTFFILCFVCKPSNGTSLVKACLVENGFGASGKDLEIVKAVANPEYVGILRQVPKDKLSGVFACIFQD RNPTTNLYASLKRLIEMDDKVSKAEAQKMQDSLTDCHKEAGDDAKLLNCVNAFDSPFDEIMATIRDVPDSLGFCYE KCKLTISEMYKIEQNRLNLKEIMTFVPEEKMACTTGCKVDKSGRSLTSRLTDLIHKSKKHDEKKKKEMIETLNRCSAQ VSGVKVETYNLIKCLNLYKPPFIDLY

NvitOBP39

MKTFTIILILGKAYCQFGNASRLEDMKTCLVENGFTDSHADLERLQAIGDPEHVDRLKDVSREKMAGVIA CMFQRRHGNKNLNEALQNLVERDDKVTEEERKKMLETLKSCNANATGDNIKLLSCLNIMAPPFDVLIAAFRDLDDS VGICFPRCNVTIGEMYKMQKNMEMKTKVKDLLKIVNEQKMGCFMTCIVDEEAKINRLSPPFLRVLTDRINKSENH DEDQKREMLKTLSKCNAQVAEVKDKTYEMIKCVNMFKPPFIDIY

NvitOBP40

MKTFAIILILGFAWRVSNASKYENMKTCLVENGFTDDETDLELIRAIGEPEHVDRLQDVSMEKMAGVMA CLFEKQQGNGNLNNALESLVGRDDKATEEEKRKMLETLKTCNTNAAGDNTKLLSCLNIMAPPFDVLIASIRDFDESV AVCFPKCEITIGEMYKMEENKSKVKGLLEIVNEQKLACFMACIVEEEEKNRKSPHFLKALTDLINKSEEHDENQKKE MLETVDKCNAQVAEVKDKTYRIIKCVNMFKPPFVDLY

NvitOBP41

MKTFVFILLIVYVCQWSDARETDFEDLIFNDLMSMCLIENGFTNSTADVERMYAIGDPEQADKLKDVPKE KIVDVIVCLYHKLYPYRNLYPSLRQLIDRDDTVTVGQIRQMEHTMSDCYKEVGGKINNAELLKCVDITEPPFDHLFAA IRDVRQALQWCFVRCGVLISEMYTMEKNRDLPFKEYIKYIPHERISCLMACKAEQAVVNNSDQTLQKGLADLIKRSK NVDEIEKAEMLKSLKKCSKKVYGYEDEHYELVKCIDLFKPPFIDLF

NvitOBP42

MHYAIFYICITFAIILVLAFVWQPITASFRVKYSETCLMENGFTDSPSVVACVYQKQYADKNLNNALKVLID RDDKVSEDEAKKMKETLATCNTNAAGDNAKLLSCVNLVAPPFDTLIAVIRDLPESQDPCFLKCDMKIGMPMNIQII TISSVITENGIRLSDSHFSGEMYKAEQNRNKMKDLVKHVPEEKLSCIFGCKVDEFDKTKPGGKVHDENHKKEMLETL KRCTDTVAGQPNENTLLGKCLDLFNPPFVDIY

NvitOBP43

MKTFAVVLVIGFALQVSNALMPSDLETVVETCLVENEFTSSKDDVALVDYIGKPEGVDKLKDVPREKLAAV LACMYHKQYKNTTLYDMLKYLTEMDDKVTKDQYQQMKETLTTCVQQCNGINLIRRDYPQGNDVELLKCVKAVDP PFDYLIATLRDLGEEPLNFCYERCGLKILIRILIIRSLRTLHTRDY

NvitOBP44

MKLHFYAMNTLVSFFVLCFVWQLSHAIAFSDLTTCLIENGFTNTTSDLKRIYAIGDPKQADKLKDVSKEKM VNVVACLYDKLYPHQKLYPSLRQLIDRDDTVTSDQIEQMENTASACYRKVDGKINNVKFLNCVNITEAPFDHLFATI RDVQQALQWCYVRCRILISEMYTLQRNRYNPLKDYIQYIPTDKLGCFMACKAEESTMKNPDQTLQKRLTDLINLKK NIDESKRKEMLQTLNKCLTQVPADEDERYYEVIKCINLFQPPFVDILYNNFYSAYNIEKKELNI

NvitOBP45

MKTFAIILCLGFTSQVSHAFFPPDVEKCMVESGFTNIPDDLGIIDGVGNPNDTDKLKDVPKEKLAIATACM
FHKEYTNTLYDMLKLLIGMNDKATNDQRQQMMDTLNTCHSEVKDNDAELLMCVKAFEAPFDYFIETLHMFGKSA
LYPCYGHCGLKIPDLYILEEHRNHVDELLKRIGTDNVYCITACKVERKEDNPALALYTMLSEIIDRRLKDDKNTEAKAA
LEKCYTDGEYQKVEMAGSLGETIPKGSKVRDTYQLLKCMKVFEEPWINLMRN

NvitOBP46

MKRFLILFIFCLTSPLGRADIYDDTVTCLKERGLKETDIRILDAVGEPGNEHILKDAPKDKLVDAIACIFRKDH
KTNKALFGTLTELINMDSEIDNNKRKELLVTLNACNKKTDGNDTNLLKCLEATQPPFDKYAAIIRDVTNVIKECFFSN
KLTFSDLRYRKMLHRESVSEVLKHVGEDNITCAMTCEFDKNKHVYKDMDPNETIHKRLETLITKSSLPEEKKREMKE
TLDRCDHQARGDSCKFGKCVKLMRPPFVQLYFKNP

NvitOBP47

MKSFLIIQLICLSGLLRAVNAESVTTCLVKNGIKSIDTATFEEVVKTNGDALKTVDKQVACVIACAFDDFTQK
KESIYNYLTRIIKKDLDDEYSASERKEMLDILNYCNGLAFGNDCKFLHCINVTKAPFADLILTVGIEDSGKGEDQKMF
M

NvitOBP48

MKNLSIVLIACFAVFQLTSADSNHIKECLTEHGLQETDMNLLKSLGSVKFADTTIKDEDREKLDCVLACIFQ
KESPGQSLHEVIKDVLSEDTYKSENLRKEMTETLNTCETQAGDDDCKLLQCVQITKLPFYDLLYTFVVARDMQKCFV
DNGLNASDAVNLEHLNEPGWVEEILKKVDEDKLACTMFCLYSKQDKITHDKSLQQRFETRINESDKLIADQKKEML
ETLNRCGIEAAGDNCKLVKCIKILRAPFFNLYHQ

NvitOBP49

MKRFIIIVCFYFIYLHTSSARRSDNLKKCLVEHGLRNKLYKRAIGGSNINSLDRDVNVDLKEKLACTLACSFRK
EHSGNETLHAIFTEALNFGNFHILEDVKQQMRETLNNCYTEVESNDCKLLHCIKIIESQFMDVVLAITNERPVRLSFE
NTNSVKKFKKIKFRIKWFKVMHMMIRSLQRHIPYKQ

NvitOBP50

MKRSLVVLSFCFLLLQSVRIVADDESEELKECLQDNGLPTNIHKEVGKGEFEKLKDVPESKIACVIACSFNKR
IRSNDFLFTSFVKAVNKDKSLGEDQKKEMLETINRCSKEAKGDNCKFLDCLKITKPPFVNLIITPKDANDKKI

NvitOBP51

MNRLFIFLIVLVAGSRADKRHDYGQKMSQCLIENGLNETHLKYVFRIGKVNLEIPSDVSEEALACMLACVY
NFTITMRETQKTLDEAILDVINLDDMFNNDENKRKALKETLDKCKTEAAGDNCKLLQCIKVTRDPFDKIITAGRYNY
GYLRRDNE

NvitOBP52

MKNLFVILALCVVSLYALDGFDFYRKKMDECLSENGLSEYDLKYVFKIGKPDYESPKNISDKTLACMLSCMY
HLYKNRQTIDKAIATVINRDDSLTAEKKKNLLGTVRNCTREAGDDDCVLLHCLDVTKEPFSKIITAGKKIKPSKIDTD

NvitOBP53

MKILLFLLIFCVVGIYTQKHNDSAEKNKHAMDIEDCLNQHSNITKKGLSVKDIILKSIAPYDLGCITSCLKKKE
LKNGVTLNSYVIQNAYLPSTKFPDWYEKKNEDYQYVVIANRCINEAKEDECKLFMCLKAWELPFAHILSIKIERYITF

NvitOBP54

MKLCWIILIALCIFGINARPNSEPDNDGGFEPLALQCLRELKKDPTLSAKNCDEIESSLTDDERNCILACMFR
RNDPDKKSLYEYLKSQLSTIDNRIQVYRDELLEKLNSCKALVGEGNDCGVMKCIELFKPPFAHWYLQTN

NvitOBP55

MRNSLVVIVLICFSQIHAIPLTKKKEKFVPAEDSKEQCMIKFGLDPDFVDYLIGLHRPQIEINAYIGSKHSCIH
ACMVKLDQNLNPYDYVVDRVSADTKEEYERLIKLVNKCNEKDSGNGCVLLDCVRRNKELRDFVY

NvitOBP56

MKLFVFCVFALCLTAANALFGPKLKEKLLEREDACLRETGNTLLSIDHVRRTKTLPEDGSLDKFALCLLKKHR
IVNDDDTVNKDKHRYYLILDDGRKKEYAEDCVLSSGGSNNGEIARHLLSCLLKTDIFFIDWSYRSQEVLSQMRQRQK
QKTQA

NvitOBP57

LTFNFRTNTDILFQASTGNIVNDTLNRKFLLLVKTCANKKLHMSDYGSINEDVNSCHLIFDNSSMLETVEKC
KNRRETASRDMTCFLKSHVLIIDPYIGAHESA

NvitOBP58

MKFFISCVLVIFCSSSAIGLLSHEAILSLQRDQDDCVRESGVTRSTVEQAHLDRVIHNDENMAKFAACMLK
KFNVMSDDGKINEDVYSYHLISDNPAMFETAEKCKKRTGSDVDETASKIMTCFLNSDVFVLDPYIGVHKRA

NvitOBP59

MKFYALCVILLCSSAAFALLEARVRDYLYEYQRDCMIESGADTSLVAAADRARIIPNDGLLDTFAICMLKKY
NILHKDGSVNQDHDSYTIFSDNPDVYRISERCKAKIGKDAGETARKIMNCFAEDGDSLLPYSTHPPPTPC

NvitOBP60

MKIYVLCAVLFFTPTVFGIYSSAIWDALLHANEEPCGRSAGLSEESIESSRRARYLPESPEMNVFAFCVIRVL
NIMSKDGKVNPDIGSYLVPTNTPDITKVISEKCRTHVGVDAGDTARTILNCYLQADQLVISLPSDAQLTFN

NvitOBP61

MKIYVICAVLLFAPAALGLFSNGIWDVLHANEAKCQLNSGASDASIEDARRARKLSESPEMNAFAKCMLG
IYNVMRPDGSINPDFQSYTVPTDVPNNTWRISQKCITLGGTDSGDTARKIFNCYTENNQLVMAWTPKVSV

NvitOBP62

MKIYVICVVVFFLAPAVFASFSPLIEDDFHAYEADCGASDESIEAARRARQLPQSPQMNAFALCMMQKYK
VMAADGSVNPDVRSYGIITDGPDNTWRVSEHCRTLNGNSAGETARMIMNCYLDNNQLVMGLTPRVSA

NvitOBP63

GTFLPSIDDTLHEYETNCARVSGATHSAIEIARNTKMLANTARLNAFAMCMLQQFNVMDSNGIVNPDV
MSYSIISNVPNATAGISQQCISKRGIDAVNTARMIMNCYLRANQMVLALSRRDCT

NvitOBP64

MKKFTLIFVSCYLVFSSMHRVMCVTQCFFNELNLVDQRGFPERSAVIGIMTQNIQDPELRDFVEESVIECY
HYINNNNSGRQEKCQFSQSLLSCLAEKGSERCEDWDDE

NvitOBP65

MKSILFIFAIVCVVGVFSDDDKKDLTREQILECVAESGVDETKVEDIKLGNQGLETTREIDCFAACVFKKQGI
MNEAGVITPDKPMDNEAAKQCVATTGADACDTAGKVLKCFISNNLVSLMDLDDD

NvitOBP66

MKSVLVVFAAICIAGVLSDPKGDIDACVAESKVDTKLFEDMMHTPDFKATREMDCFAACMFKKDGVLD
ADGNVDASKLPNVDVSKVCGALRGKDACETAGKIIGCFAEKGVMDVFHIV

NvitOBP67

MKTSALLLVAFGIFAFTELSTASLDKWFEECVKSYGHTEESVSKLPDLEKSCVIHICFMRDVGLINEDNSLNV NYLLERRKSHVPESKIYDAVRTCNAESIDTLAKTCEAVKCLMDLLHESDFNTQPNVTD

NvitOBP68

MFAFAVFAFTNVLNPMYFHTFYETTFFLSCVESIFKLTESERSCAFQTSFLRELGLINKDNSFNVNDLLKQR KSGIPESKIHDAVKTCDVESLDSLEKTSKAVKCLMGLLRNMWLM

NvitOBP69

MKLFAVVLVFFALGSSSVALDEEERGVLRQIRNVCVVESGLSPYELGFIYRAIRPAKKLAQASRCVIQKISEL QSENETVKHIADRGKAALANAPISNIADNVLGSCQNLLGQNGCIQVLELAAKIIDNLRSRRQ

NvitOBP70

MISGEEISLLVIFTICWGINLKCKHAGEIQLHLQDKEAAEKCSKDIGITLETVYATMKNELKDADEKLKCFAA CVFKEKEMLKDDGPINVAKAIEDLPDEIKDDVRDAMIKTIEKCSQKKEANECETVFHAVQCATLDMSKLKLF

NvitOBP71

MKVAIVACVLTICSIFAGSKADLTEDQRKILQPLKDECFQETGLDAVTLEKFKKEALQKFKTTGEVSNDEKV NCFSACMFKKIGFMSEEGKFEEDTVRALMSENFPPETLDKAIENCKNEVGKDHCETAAKLIVCFMNNKAGMENV

NvitOBP72

MLFFTVVLLFSSVCTATKEEEEFKSELAECKNLVGVTEDYVRDVFKSGLKGADEKFKCFIACLIQDSYKFND GGVFDAERTIANDRGPAGLLRDYTNKALKACSNIKGYSECDAIFKVYKCMVENVEKLFNARNDRPSG

NvitOBP73 MKDDGTLDIEGTSMIVGRRLKYAERNDVIKADQACSNIKGDNACDTIFKIVGCSIKNLERYR

NvitOBP74 MMDIEGSSMIVGRRLKDVERNDVIIAVQACSDIKGDNACDTIFKIVGCSIKNLKRYR

NvitOBP75

MRVLLVVVSVCFVGSYADYADDIRKLQEETKRIEEYRRPCLKEVGLYADPANGITSQPASSPTIGQIFCLWA CLYRKNGSIRPDGSVDEAAVRSKNPELEGPLDVIISKCENQAGENTCKLAGCLAKAHFNLLE

NvitOBP76

MMQGSLCALVVLSLVCLVRAGPPDWISAEILEMVQSDKGRCMAEHGTTEALIDDVNKGNLPNDKAITC YMYCLFEAFSLVDEEANIEVEMLVGFLPEHMQAVANELIDVCAKLDGADVCDKMYVMAKCVMEKRPDLWFML

NvitOBP77

MKIVVLCLVVLSAVACVSAGYREYQNACLDENGLTKEEFYAMKRNQDPRSGCVTACIMKKNGSMKHGII DARGIKRRMRTLLAPFISKDKLYEKIDYCVDEAENHVGVCEKAYVLQKCLRTPRANNVQGERQKMID

NvitOBP78

MKTIVFTLCMMTVAVTCSPRPGGRGGGSMFSRESVKKCMAEMDIKREDIKTLKQNNDPKLSCLNACA MTKEEIMDEAGNIDADKLIKATLEIVQKKKPDINVEELETAMLSCIEKAKEVEDKCMKAKTLVVCSHEYWKANVKG NPSSAGGEEE

NvitOBP79

MKLFFVTLCVLFAAVYGATKSDSKSEKIFHECLEENDIKESDFKNLEGKKDPKMRCLMACILEKEGALKDGE IDGDVIKKDIIAEFTEVDAQKISDAIDTCVDGANDLSDICEKTSFIGECLKVELDKLEMNMN

NvitOBP80

MGGFVTVLYFLSIIICVYSLNWSEAKKHVQECLDEYQITREDVAKLKKEESPDYNCYIACIMKKRGSLVDGKIDEEKMLEILKQLHVLNSERTEDKFRICATEANKQSNECLVAGDMIGCLYFKSN

NvitOBP81

MKVIVLLVTVLTITIHVSCQTDEEVHKIKEKCFDLSDIPVEDRVVYNPENPKLKCFNACTYTGVGMMKDGKIVPEKYIERLQDSLKNEKKSDVEAFMKHMEDCAVMANKLSDECEVAYSMIKCL

NvitOBP82

MKRVMALVGAFLLVSAVQCDDMPFWNEKVECAQSMGISPDQMTSMLTSNDAQMNCVHACVLEKIGGMVDGKLSLDSLMESLEKLKAEVKDYDATKAGIHQCFDQASGDRCESAGKFAMCMQEHMQG

NvitOBP83

MRLTLQLITLVSLVAIFKTTESKMTMDQIKNTLKPFKNSCIKKISPDVAMVEATKSGQFPEDATLMCFLKCVLSMMKVMKNGEILLPSIMQQIDIMMPDEYVETMKEICTNCYEMSLKVDDACEKAYVFVKCYYNTNSELYFFP

NvitOBP84

MRRSILITSILIILISQYKLVKCKKMNLDELRDMLRPMSKSCKSKTGVSDEMVAATHQGIFPREKPLMCYFKCLSVMLKVMNKQGEIKPKDVERQIDLLVIPELAPTLKKIGTDCYNKVAPTNDACAYAFEIVMCGYQTDPKYYFLP

NvitOBP85

MRSVLLIFCLSSVAVRVSAHVSPVADSFKACLAESGMTRDDFIKALQSSDDSKAQCIAACTMEKEKFMSDDKINVDAIIAKMEDVSQEIGKVQITDLVMNCAAEAKDKSGKCGVAHSVVRCIHEELRKEGWI

NvitOBP86

MKSYSVILLAICFAAIYSSSALISIEDKAACLKKNGLNNTEKWDLTAQFDYRLEKPFTCYVACVINAIKKPEETVYGKLSEVIERGHVIPASLKKDMENRLDSCYRYNGEGDDCKLLYCVKILQSPLIKLSIYSLEDIEL

NvitOBP87

MKFLIFVISLFTVVARSRQSLADIEACASQYGVENVTRIPDNDRPFKQRDPDYECLRACLWRKQGIMKNGKFDLDKAFNYFKKTTRFPLTVFKEKLSVCVEKGNQEKNECGVTRVYVDCMNGSPKARK

NvitOBP88

MKLLIFVISFFIVAAHSQPRSMDWKGCMEEIGVSKDDVKSTEWGDPKSRCVLACTFKKVGVINDGKVVFDVAFDITKGEAQDSSHDKYIEEKVNSCIEKAHQETNECDVSYVFMECMKTNNNTAKMANGTMSI

NvitOBP89

VTTNNDLFKIKDAKVVSNVAFDMAKEDTRSSSDKDTQEKVNMCIEKAHQEANECDVTYVFLDCLADGLIMAKKQVVNIK

NvitOBP90

MKNLALLLLTLCVVSCLLINGARAGVSREQMEKMANGFRNTCVGKTGADMSLVEGIRVGNFVEDPTSMCYTKCIMGLMKTFTKQGNIDVEMLVKQINVMASPDIAGSMVTNARKCHAETSASDPCELAWLFTKCIYAADPAVYFFP
