# Supplementary material for: Unique Features of Odorant-Binding Proteins of the Parasitoid Wasp Nasonia vitripennis Revealed by Genome Annotation and Comparative Analyses
Source: PLoS One. 2012 Aug 27;7(8):e43034. doi: 10.1371/journal.pone.0043034 (PMC3428353; doi:10.1371/journal.pone.0043034)
Supplement: Figure S4 — The Coding DNA sequences and EMBL entries of all 90 Nasonia vitripennis OBPs. (DOCX) [file pone.0043034.s004.docx]

**Supplementary Figure S4.**

>NvitOBP01(EMBL: HE578186)

ATGATGAAGAACTTGACTCTGTGCTTTTTGGTCGTGGTTCTCGGCGTCATCAAAGTTAAC

GGCAATGAAATCCCTCACGAAATCCGACACATGGTGGTCGGAGTTCGCGACAAGTGTCAC

CGAGAGACTGGAGTCGATATTGAGCACGTCGACAGAACAGTCGAGGGATATTTCCACCCC

AGCGAACTGCTCGGATGCTACTTCTCCTGCATATTCAACCACTTTGATCTCCTTGACAAG

GACGGTCACCTCGACTGGGACAAGTTGGTCCCGAGGATCCCCGAATCTTTCAAGGAACAC

GCCGATGAGATGATCGCGGCTTGTCGCTCTACAACCGGAAAGGACCCCTGCGACTCCGCC

CTGAACATCGTTCAATGCTTCCAGAAAACCAACCCCTCGAAATACTTCGTCATC

>NvitOBP02 (EMBL: HE578187)

ATGTCCGGCCAATCGCTACTACTGCTCGCGCTCGGGATATTCCTGCCGCACTGCCTCGCT

GGGACGCGGCCCAGCTTCGTATCCGACAAGATGATTGCCACGGCCGCCAGCGTCGTGAAC

GCCTGCCAAATGCAAACGGGCGTGGCCACTGCCGACATCGAGTCGGTGAGAAACGGCCAG

TGGCCCGATACCATGGAGCTCAAGTGTTACATGTACTGTCTCTGGGAGCAATTCGGACTG

ATCGACGAGAAGCGCGAGCTGAGTCTCAACGGCATGCTGACGTTCTTCCAGAGAATACCG

GCTTATAGAGTCGAGGTCGAAAAGGCTATTAACGAGTGCAAGGCCCTGGCGACTGGCGAT

ACCTGCGAGTACGCCTACACGTTCAACAAATGCTACGCCGAAAGATCACCCAGAACTTAC

TACCTCTTC

>NvitOBP03 (EMBL: HE578188)

ATGAAGAGCCTGCTGCTGTGCTTCTTGGTCGTGATCCTCGGCGTCACCAAAGTCAAGTCC

AATGAAATTCCCCAAGAAATTCAAGCCATGGTAGTCGGAGTTCGCGACAAATGCCACCGA

GAAACCGGAGTCGATATTGAGCACGTCGACAGAACAGTGGAGGGATACTTTCATCCTAGC

GAGCTTCTTGGCTGCTACTTTTCCTGCATCTTCAACCACTTTAATCTTCTCGACAATGAT

GGCCACTTGGATTGGGTGAAAGTAGTCAACGTGATTCCCCCCTCGTTCAAGGATCACGCC

GACGAGATGATCGCGGCGTGTAAAACTACGACTGGAAAGGATCCCTGCGATTCGGCCGTT

AATATCGTCCAATGTTTCCAGAAAACTAACCCCGCGAAATATTTCGTCATC

>NvitOBP04(EMBL: HE578189)

ATGAAAGCTGTCGCCATCATCCTTGTGGTTTGCCTCGTTCAAGGACTCCAAGCGCTTAAC

AAGTCGGAAACACCCGGCTTAAATGACCAGATGAAGGAGTGTCTTACTCAGAACGATTTG

GATGCAGATCTCTATACCGAACTCTGGAAGGACCATCCTAAACTAAACGCTCCTCAGAAG

AAGGTCAACTGCTTCCTCGCATGCCTGTACAAGAAAGTGGGAGCCCTAAGCGCTGATGGC

GCGATTGTTCTTCCTGAGGGACTCATCGAGGAAAGGATCATCAACTGGTCTCCTGAGCTG

AGAGAAAAGTGCAAGAAGCAAGCTGGTGACGACGTCTGCGAGTTGGCCGGCTGCTTGGAC

AAACCCAACGGATTCTTATCGGCAACTGTG

>NvitOBP05 (EMBL: HE578190)

TCGTCCGCTATTTCACAATTCGGTAAGCTCAAAGACGCGCCAATCCCTTCTCGCCAATAT

TACCTTCGGCATCGGAGAAACAGAGAGAGGAAAAAAGGTGCAGGCGCTCTCCGCATCGGA

ATCGAATTTCAGCGAGATTCATTGTGTTACGCGCTCTGTATCGCGTTTCTCGCGGCCGAC

ATCGATCCGACAGCGGAGATCGAGATAGATAGATCAGGCTCGCGTGCGATGCGATGGATC

GTCGACGAGGATTTTCCGCGCGAGAGACACCGGCCGACCGCGAATTGCGTTACCCGCACA

CATAACGCTATAACCTCGTGTCTCGGGTCTTTTCCGCTCGTGCGGTGCACT

>NvitOBP06 (EMBL: HE578191)

ATGAAGTTTCTCACATCTGTCCTTTCCTGCTTTGTTATTCATGCGATGCTCGTCAGGTGT

GCCCCCTTTCACGAGACATTAGATGATGATCCAGATTTAAATGACAGTATTGATTTATGC

GCTGCTGAAGTTGGTTTATTAGTTGAAGAAACCAGGAAAAGTTTTAATATGCCTATAGAA

GCTCCTGGAAATTGCGTAGTAGCGTGTGTGTGGAAAAAAATTGGCCTGATGGAATTAGAT

GGAAAAATTGTTAAAGAAGAAATGATATCGAGTCTCCATCCGACTCTCAAGCAAATGCCC

AACATAACTCCAATCCACGAAGACGACTTCTACCATTGTGTGGACGAAGCTAATGATTAT

GAAGGTGGCTGCATAGTAGTCTCGGAATATTTCAAATGTATAATAAGGGATTTATTTAAC

CAACTGTCTATA

>NvitOBP07(EMBL: HE578192)

ATGAAAGCTTTTCTGTGCACCTTCAGTATCGTCCTTGCTGCGGCCATGTCCGTCAATGGC

GATATGCCAGGCGAACTGAAGCCAGCTTTCCAAGAGTGCCACAACGAGTTGTTAGGAACG

CCTCAGGAGGAACCAACCGGACCACCCAACATGGACGACCCCAAAGTAAAGTGCATCCAC

GCGTGCGTCGCGAAAAAAATCGGCCACATGGTTGACGGCAAGATAGTCGCGGAAAAGGAA

ATCGAAAGCGCCAAGCAGCACATGCCCAATGCTGACAATAGCTTGACGGACAAGATCACC

GAGTGCGCTAACAAAGCCAACGAGCAAAGCGACGAGTGCGAGGTATCTGCCGCCTTTCAC

AAGTGCATCGTGGAGAAGGTCGGACCCCCAGAGCATCACCAC

>NvitOBP08(EMBL: HE578193)

ATGAAAGCTTTTCTGTGTGTTTTGGGCGTCATCATTGCCGCGGCGTCCGCCAGCTGCGGA

ATGCCAGAAGAGATGAAGCAGGCTTTCAAAGAATGCCACACTGAACTGGGTATGCCCGAT

GAAAAACCTCATGGACCGCCCAACCCTGATGACCCTAAGATCAAGTGTTTCCACGCATGC

ATCATGAAGAAGGCCGGCAAAATGGTTGATGGCAAGTTGGACGCCGACAAAGAGATCGAA

TTCGCCAAGAAACGCATGCCCAATGCCGATGACAGCATGATCGAAAAGATCACCGAGTGC

GTTAAGACAGCCAACGAACAAAGCGACGAGTGCGAAGTGGCTGGAGCCATGCACAAGTGC

ATAATGGAGAAAGTTGGATCCCCTCCTCATCATCATCGCCAC

>NvitOBP09(EMBL: HE578194)

ATGAAGGCCGTCGTCATCGTTCTTGCCGTCTGCCTCGCCGGCGTCTTCGCAGAAGACCCC

ATCAAGGACATCAACAAGGAGTACATCAAGGGCTGCCTGATCGAGAACGGATTCGACCCA

CAGCAATACCCGACCGGTCTGAGGAACGCCAAGGTGCCCGAGAAGCAGGAGCAGAACAGG

AACTGCTACTACTCCTGCATGATGAAGAAGATGAACCTGATGAAGGCCGACGGATCCCTC

AACGAGGACGCCCTGCGCCAAAAGTTCAACATGAACCTCGACACGCTAGGAAAGGCCCTG

AGCACGTGCAAGGACCAAGTTAAGGACGACAAGTGCAAGCTGGCCGCCTGCCTGATGGCC

AACCGTGGAGCT

>NvitOBP10(EMBL: HE578195)

ATGGACCGGCACCTCATAATAGCATTGACGCTTTTTAGCGTCGTATTCATGGTTCAAAGT

TTGAGTCAGGAAGATATAGACGCAAGGAATAAATGTTTAAAAGAACATGGATTTACAATA

GAACCTAAGTACGTCAGTGCATATAAGACAATCGACATTAGAGCGAAATGTTATGCGTCG

TGTCTAATGAGAGAAACTGGAGTAGTAAAAGAAGATGGTTCAATCGATCTTAATAAAGTT

CTGGAGAAAATTTCCGACTCTGAAAATAAGACTTTGGATGAGGTTGTCAAGAAAAGTTTC

ATACCATGCACAGAAAAAAAAGGGGATAACGATTGTGACACTGGCCATCAGATTCTAACT

TGTATAGTGGCAACTATATCCATCTTAAAAGAATCAATGAAGATAGTT

>NvitOBP11(EMBL: HE578196)

ATGAGTCGGCATCTCATAATAGCATTGGCGCTTTTCAGCGCCGTATTCATGGTTAAAAGT

TTGAGCCCAGAAGAAAGAGTCGCACGAGATAAATGTTTGAAAGAAAACGGATTTTCTAGA

GAACCTGACTTCATCGGTATCGATGCAGTCGACATGAGAAGCAAATGTTATGCGGCATGC

GCTTTAAGAGGATACGGAATAATGAAAGAAGATAGTTCAATTGATATTAATAAAATTCTG

GAGCACATTTCCGATACTAAAAATAAGGATATTGATGTCAAAAAAAGCCTCATAATCCCT

TGTGCTGAAAAAAAAGGGGAAACCGATTGTGATACAGGATATCTGATTACAAATTGTGTA

GCATTAGCTGTGAGAAAATTGGATAAA

>NvitOBP12(EMBL: HE578197)

ATGCGTCTACTCACTGCTCTTCTGCTAATCGGTATTGTTGCCGTAGTCAATGCGAAAAGT

GGTTCGACTGCGTTTACGATAACGCAAAACGATAGAAATGTGTTTCGGAATTGCATGACG

AAAATAGGAATTCCAGACGATGAAATGGTCGCTGTCTTAGACAATCATGAAAAAGATGCA

GATGAAAAAGTGAAATGTTACAACGGCTGCCTTTACAAAGCTTTCAAAGTCATTAAAGAT

GATGGGACAGTTGACACCGAAGCTGCTATAAAATTTTTCAAAGTTGAAGATATGGAATCT

GACAAAAATATCATAGTAAAATGTAGTAATGAAAGTAATTCAAATAAAGAGAAAAATGAT

TGCGATACTGCTCAAACAATGGAGTCATGTTATTATAAGTTGAAAAAAGAACAA

>NvitOBP13(EMBL: HE578198)

ATGCGGCTCTTCGCCTTCGCCAACGTACTTGGCATCGTTCTTTTGATCCACAATTCTGCA

ACGAAAACTAATGTAGAGCGGTTCTGGGATTACGTGGATGTGCTGAAAGACTGTGCGAAA

GAAAATGGCATCAGTATTGAATCTTACGCATACGCGTCCAAGAAAAACAACACAGACGGC

ATTTACGAGAAATCCAAGTGCGTGGAAGCGTGCATGTTCAAATCGCACAAAATTATGAGA

CCCGACGGTACGATCGATATGGAAAAGGCTATCGAACACTTGCTGACGGGTAATCCGGGC

GAAAAGCGAGATTTGATGAAGAAGAACATCGAATCTTGTGAGATACCGAACGGAGACAAT

GAATGCGAAGTGGCTCACACCATGGTGAAGTGTGCCTTGGGATATGAT

>NvitOBP14(EMBL: HE578199)

ATGAAAGCTTTTCTGTGCACCTTCAGTATCGTCCTTGCTGCGGCCATGTCCGTCAATGGC

GATATGCCAGGCGAACTGAAGCCAGCTTTCCAAGAGTGCCATAACGAGTTGTTAGGAACG

CCTCATGAGGAACCAACCGGACCACCCAACATGGACGACCCCAAAGTAAAGTGCATCCAC

GCGTGCGTCGCGAAAAAAATCGGCCACATGGTTGACGGCAAGATAGTCGCGGAAAAGGAA

ATCGAAAGCGCCAAGCAGCACATGCCCAATGCTGACAATAGCTTGACGGACAAGATCACC

GAGTGCGCTAACAAAGCCAACGAGCAAAGCGACGAGTGCGAGGTATCTGCCGCCTTTCAC

AAGTGCATCGTGGAGAAGGTCGGACCCCCAGAGCATCACCAC

>NvitOBP15(EMBL: HE578200)

ATGATGAGGATACTTTTACTGGGTTTTTTCTGTAGCATATTTGCGCTTTCTCATCAGGAG

ATTCCAGAACCCAGTTATACACACTGGCAGGAACATCTACAATCCTGTCTCGACCAAACT

GGACTAGATCTTAGTATTTTTGGTGTGTCTCGGATAGACGATGTTACGGAACAAAATCTC

AAGAAACTCGCTGAAGTAACTGCAGATAAAAGGGGCTGCCTGGTCGCGTGCGTTTTCCAA

AAACAAGGGATGATAAGCAAAGAAGGCGTCCTTCAGGCCAATCCACCACGCCCAGATCCT

ACAACGAAATTAGAAACCACATTCGAAGAGGCCATTGCAGCTTGCAGATCCGAGAAAAAT

TTCTGCAAGCTCGGGAACTGTCTATTTGAAATCTACTTCAAATACAAA

>NvitOBP16(EMBL: HE578201)

ATGAGGGTTATACTTCTATCAAGTATTTTACTCGGTAGCATCGCTATCTCTCGTCAGAAT

CCGGTTACATATACAGATAAAGACAATAACGTCATAATCCCACCCTGTCTTGCGGAAACT

GGGCTGAATCTTAGTGTCTTAGGCGTAGCCAAGATCGAAGATGTGAGGGATAGCTCCTTC

TATAATCTCAAAACTCTCACCGAAGATAAAAGAGGCTGCTTCGTAGCCTGTGTTTACAAG

AAACTGGGAATCATAACGGAAGAGAACGTTTTAATAAACGATCGAGTCATTCCACCAGGA

GTAGCAGTTCCTAAGAAGAAATTAGCGACTGCCTTTGAAGATGCTACCGAAGCATGCAGA

GCTCAAAAGGACTTGTGCAAACTTGGAAACTGCCTGTACGAAATTTACTTCTTC

>NvitOBP17(EMBL: HE578202)

ATGAAGTTCTTTACCGTTGCCACTTTCGCCATGTGCATCATCGGAACATTTGCTGCATTC

ACCATGACGGAAGAACAAGCGAAGGATCTGCAAGACAAACTCGACTGCATCAAAGAAACT

GGAGCCGACATCGCTACGCTGCTGAATATCAAAAACGGGATTCCGACACTGTACGACGAT

AAAGTAAATTGTTTCGCTGCTTGCATGTTGGAGAAATTCAATATCATGAAGCCTGACGGG

TCCATGGACGAAACTGTCGCTCGACTCAGAGCATCGAAGAGTATGTCTCAAGAAAAAGTA

GACCGTGTTCTCAGCTCCTGCAAATCCGAAGTTGGCAAAGATAAGTGTGAAACTGGAGGC

AAGATTTTGGAATGCCTCATGAAGAACGACGCTGTTCCCATCCTCAGC

>NvitOBP18(EMBL: HE578203)

ATGAAGTCCTTCGCCGTTATCTTCGCTTTCTGCTTCGTCGGTGCCATCGCTGCACTCACG

GAAGAGCAGAAGGCCAAGTTGAAGGAATACAAGTACGCCTGCATCACTGAAACTGGAGTC

AGCGAGGATGTCATCGAAAGCGTCAAGAAGGGAGAGCAAGTCACCTTCGACGAGAAACTC

AACTGCTTCTCCGCCTGCATGCTGAAGAAAGTTGGCATCATGAACGCTGACGGAACCGTC

AACGAGGAAGTCGCCCGTGCCAAGGTCCCTCAGGATCTGCCTAAGGACAAGGTCGACCAA

GTCATCAACACCTGCAAAGCCGAAGTCGGCAAGGATAGCTGCGAAACCGGAGGAAAGGTC

TTGGCCTGTCTCATGAAGACTAAAGCCGTATCCGTCCTTCAC

>NvitOBP19(EMBL: HE578204)

ATGAGGATACTTTTACTTAGTATTTTCGGTAGCATCATTGCGTTTTCTCATCAGCAAGAG

AATCCAGAACTCAGTGATGCACACTGGCAAGAAGATCTACAATCCTGTCTCGACCAAACT

GGACTAGACCTTAGTATTTTTGGTGTGTCTCGGATCGACGAAGTTACGGAACAACATCTT

AAAAAACTTACTAAAGTACCTGCAGATAAAAGGGGCTGCCTGGTCGCGTGCGTTTTCCAA

AAACAAGGGATGATAAGCAAAGAAGGCGTACTTCAGAACAATCCACCACACCCAGATCCT

ACAACGAAATTCGAAACCACATTCGAAGATGCGATTGCAGTTTGCAGAGCAGAGGAAAAT

TTCTGCAAGCTCGGGAACTGCCTATTTGGAATTTACTTCAACTATGAAATA

>NvitOBP20(EMBL: HE578205)

ATGAAAGAATTGATCGTTATAGTCGGTTTTTTGGTTGCGGCAATGCCGAGTCCCGCTGCT

TTTCAAATTGATTCTTCCAAAAGAATGAATCAAACCGTAACCGAATGTTTAACTGGTTAC

AATATCGATCCAGCGGTGCTCGATATAAATACAGAAGACGTCCACTTAATGATGGACGAA

CTTAGCGGCGAACAGCGAGGCTGCGTCACCGCGTGCGTTTACAAGGGATTCGACTGGCTC

AAGGACGATGGATCCTTGGATATCGATGCCCTAACTATGGACGAGGATCCAGAAGATACC

GCTGAATTCATCAAGGATATTGTCGATTGCAGGAATAAAGTCGGTACTGAAGCTTGTAAA

TTCTTCCACTGCCTTGATACTAAAGGTACT

>NvitOBP21(EMBL: HE578206)

ATGAAAACTTTGTTCTTTGTCGTTCTTGGCCTTGTCGCCGTATCTGCAGCCGTTCCGGTA

GAACACAGCTTTCCGCAGTTTGAAAATGGTACCCCTAAAATGAAAGAACAAGTCAACACG

TGTTTAAGAAACTACAAAATCGATGCAGCTGTTCTTGAACTAGGCGACGAAAAAAATTTT

GAGAAGACTGATAAACTTACAAAACTAGAATGGGGATGTGTTCGAGCTTGCGTTTACAAA

GGAGCTAACTTTATGAGAGCCGATGGATCTTTGGACATCGAAGTTTTGACCGACGGTGAT

GAACCCGAGGACAAGAAGAAATTCGAAAGTGTTGTGGGTATCTGCAGAGCTGAAGCTGGA

AAAGATGACTGCAAGTTCTTCCAGTGCATGGATGAGAAAGATGATTCA

>NvitOBP22(EMBL: HE578207)

ATGAAAACTTCACCCGCCGTAGTACTAGCTTTATGCTTTGTCAATGTTTTTGGGAATTCC

ATAACGGAATTAAAAAATGGTTCGTCTAGAGTGAAAGTAAATGTAAGACAATGTCTAACA

GACTATCACATTGATCCTGCGGTCCTTGAACTAGATATCGACAGGAATGATGATTTATAT

TCCAAACTAAGTGAGGAAAAAAAGGGATGCGTCACTGCTTGTGTCTATAGGGGTTTCAAC

TGGTTAAGACCAGATGGATCTTTGGATATCGATCTGCTCTGTGAAGGTGAGACGCCAGAA

GAATCCGAGGCAGAGAGAAAGAGGTATACAAAAATTGTTGCCGAATGCAGGGCTGAAGTT

GGAAAAGACGACTGCAAATTTTTTAACTGCTTGAACTTAAAGGATTTG

>NvitOBP23(EMBL: HE578208)

ATGAAAACTGTCATTGTTTTCTTTTTCATTCTCGTAGGAATTCTTGCGGAAACGACGACT

AACGTTGATAGCAGAGACGATGATATGACCACGTGTCTTGTTGAATATGGACTTGATCCA

GGTCCTAATAACCCCACTGAAGATCAGAAGAACTGCTACTTTGCTTGTATGTTTAAAACA

ATAGGATACATGAAGAAAGATGGATCATTCAATTTGGATTTAATACTTTCCGACGCATAC

AGATCGGAAAAAAGAGTAGAAAGCAAACGGAAATTAGATAATATTGTCAGCATGTGCAAA

CAACGGGCTGGGAATGATATTTGCAAACTTGCAGGCTGCTATCAAGAACATCGAAAC

>NvitOBP24(EMBL: HE578209)

ATGAATGCCGCAATAATTATTTTGGCTTTCTGCCTTGCGGGAGCTTTGGCACGGGGCATC

ATAGATAAGAACGAATCTGGGGTAGAAGTAAATGACGCATGTCTCTTGGAGTACGGAATT

AATCCAGATCAAGTTTACAATGATGGCTCGGATGGGTCAGAGGCAGTAACGGCACTAACC

GATGAACAAATATATTGCGTTGCTGCTTGCATCTACAAAGATTATGGAATTATGAGACCG

AATGGGACGATTGATACCGAAAAAGCAGACTCGTATTTCGGGGAAGATGACTCTAGGGAA

AGGGATATTTTCTTTGCGGTTTACAATGCATGCAGTGAGGGACGAGTCGGTTGCAAGTTG

GTTCAGTGTATGTTCAGCGAATTGAAAAATCATTGGGGTAGCTCGACGTCGGATTCCAAG

AAAGAATTGAAGCCGCTGTTCAATCGACCAGATTTTATTCGGGCGAAT

>NvitOBP25(EMBL: HE578210)

ATGAAGAAAATAGTATTTATCATTTCCACATTCTGCTTTGTTATGATTCAAGGGATAAGA

AAAGAATTAAATCCTAAAAAAATTAATCTAACGGAATTCACCGAAGCTATGCAGTCATGC

GGAACTATGCTCGGTTTTGATAGAGAGTTTAAACTACACCTTTTTGGCAGTCAGGAAGAC

TACAATAAAACCCTTTGCCTTTCTTTTTGCGCTCTTCGGAAACTTAAATTTTACGTAATT

GAGGATGAGATCAAGAAAGAGTTAGCACACGTGAGAAATGCGAGACTGAAAGAGGAAGTA

TACAAAGCTCTCGACGTTTGCAAACATCTTTTAGACGACCCTTGCAAACTTTTCGACTGC

TTTTTCGATTATGCAAAAGTGGTGGATGCAGAGAATGAATCTCTGAATTTGTCAAAATTT

TTCTCCGAAGGAAGCCTA

>NvitOBP26(EMBL: HE578211)

ATGAAGACCTTCGCTATTGTCCTGACTCTTTGCATCGTTGGCGCTTATGCGTCAACCTTG

AAGGATGACCAAAAAGCCAAATTGAGGGAGTACAAGGAATCTTGCATAACTGAAACTAGC

GCTGATAAAGCCGTCATCGACAGCATTATCAAAGGAGGGCCGATCAACCGCGATGAAAAG

CTCGACTGCTTCTCCGCTTGCATGCTGAAGAAAATTGGCATCATGCGCCCCGACGGCAGC

ATCGACGTAGAGTCAGCTCGCGCCAAGGCAGCCACCACCAACGTCGACGTAGCCAAGGCT

AACGAGGTTATCGACAAATGCAAAGATCTCAAGGGAAAGGACACCTGCGAGACCGGAGGT

GCGGTTTTCGGATGCTTCATCACCAACAAGGACTTCCCAGTCCTCAAC

>NvitOBP27(EMBL: HE578212)

ATGAAGAAAATACTTTGCTTCATTGTCTTCTGTTTAACCTCGAACGTTTGGGGTGAGACA

TCAGCGACTGTAGAGCCTCCGACGAAACTCAAGACTTGCGCAAATGTGACCCGGATAACC

GTAGCTGTAAATTTGCTAGATAAAGAGTGCATGAAAACATCCAGCAGCAGTGCGATTTTG

CTGAACGGCGATGAAAACAACGTCGAAGTGAAAGATATTGAAATGAACGTGTATGCACTT

TGTCTGCTGCAAAAATCTAGCATTATGAACGAGCAAGGAAAAATCAACTTGAACTTTGAT

ATTTTTAAAATTGTAAAAAACTTGTACAAAAGGACTGACCAAAGGGGCTTTGGTTTGGCT

TTTATAATAAAGTCGCTTGAAAAGTGTCGACAAACTGATGGACCTGATCAGTTTAGTACG

GCTACGAAAATAATGAAATGCCTGTTGGACAACCAAAAAACTGTGATACGGTGCGATCAG

CAT

>NvitOBP28(EMBL: HE578213)

ATGAAGATCTTCGTAATCGTGGCTCTCTGCGCTGTGGCTGTATACGCGGAAGAGAACGAA

GTGCTAAAACAGTACGAAAGAGATTGCATGACTGAGAATGGAATCGACCCAACCGTTCAA

GACCCCAAGAATCTGACTCTAGAAGATGGAAACTGTTACTATGCTTGCTACTTTAAAAAA

TTCGGAATCATTAAAGAGGACGGTTCTTATGACGTGGCGGCAATCAAGGAAAAGTATTCG

AAGCCTAATTCAGTAGAAGCAGTTCAGAAAAAGCTAGATGAAATCACGCAGACGTACTGC

CAGGACAAAGTTGGAAATCACTGCAATCTAGCAGCTTGCCTTTCTAAAATATCAAAGGAA

CAGTGGAAAATC

>NvitOBP29(EMBL: HE578214)

ATGAAAGTTTTCGTGGCTCTCGCGCTGTGCGTCATTGCAGTCAATGGCGAAGTTACCGAG

TCGTCGTCTACGGAATTTCCGTCGGCTGTTGACATATACAAAATGAAGATCTTCAAGTAC

TCGATGGAATGTCTGTTTGAACGAAAGCTCGATCTGAGCAAATTTGCCTTGCAAAAAGAC

GTGAAAAAAGCTGTCGAAGACCTGCACAAAGACGAGAAAGCTTGCTTCGCGGGCTGCGTG

TTCAAGAAACTCGGAGCGATGAACGACGATGGGACATTCAACGAAGATAAGCTATTCATG

GGCGCTACGGCCGAAACGCTGCCAATCTTCAAACAGACGCACGACGCGGCAGTCAAACAC

TGCACGGATAAAGTTGGAAAGGACGAGCTTTGCAAATTCGCTGCTTGTATCGTAATCCAA

GCTCCAGCTTATGCATCATCCCTGAACGCAACCAGTGGCATA

>NvitOBP30(EMBL: HE578215)

ATGAAGCTCCACGCGCTGCTCGTCTTGTGCTTCGCCACGGCTTCTGCGAATATCAGGCTG

ACCGATCAACAGCTCAAGGAGTACGTGCAAGTGTGCCTAGCGAAGACGAGGCTCAGTCAA

GGCTTCTACCAGAGCGGAGATGAGGCTCAGAAGATTCTAACGGAGGAGCAAAAGTCCTGC

TTCCTGGCGTGCATGTTCAAGAGGACTGGAATCATAGATCACGATGGAAGCGTCAACTTG

AAACTGGGCGACGAAGAGCTACCCAGAACACCCGCGATCGAAGCGTGCATAACGACAGCT

AAGGAAGACATCTGCAAGCTGGCGATATGCCTGCACAAAACGGGCAAGTTCAGTATTACT

AGCGTAGCTGACAGCCCGCGGTACCCTCGCTATCAC

>NvitOBP31(EMBL: HE578216)

ATGAAAAGTTACATTCTGCCAATCGCCATCTGTTTTGCAGTAATCGACATGATATTTATT

CGAGTTTTGCAATGTTCCTTTCTTCCGTTGGAAAAGATGAACAAGAGGCATGCTTACATA

GTAACGGGTTACACAATAGTAACTGATGTGGAACTTTTGAGAGAGTTGATGCTGCGTAAT

GATTCGAAGGATGAGAAGGCAATTGATTTGGAAAACAAGTTTACGTGTGCTGTTGCCTGT

TTTAGCGATGCAAAAATTAATGTGTCCCGTGAAGAAATAAAGAGTGACCTGATGAACACT

TTGGACACATGTCATCAGAAAGACGAAGGTGACAACTGCAATTTATTAGAGTGTGTCAAA

GTACTCATCCCTCCATTTAAAGCTCTACTTATTTTCGCT

>NvitOBP32(EMBL: HE578217)

ATGAAAAGCTACGTTCTCGCATTCGCCATATGCTTTGCAGTAATCGACTTGAGTTTCGCT

CTTGGAAAAGAAGATCAAGAGAAATGCTTGCGCAAAAATGGGTTGAACAATTCTACCGAT

GTGGAACTTATGAAAGCATTCGTACGTAGTGATGGGAAGAACGAATTTCATTTGGAAAGA

GAGTTTTCGTGCGTTGTTGCCTGTGTTATCGACGAAAGGAGGACTGAAGATAATGTTAAT

ACAAGTACTTATCAGCTACTCACGGATCTAATCAGTGAAGCTCATAATAAAATTCCGGAT

GAACAATGGAGAGACATGAAGACCACTTTGGATAAGTGTCATCAGCAAGACGAAGGCGAC

GACTGTAAATTATTGTACTGTGTGAAAATACTCAGGGATCCATTTAAGGAACTTATTGTT

TCGTTTGAC

>NvitOBP33(EMBL: HE578218)

ATGAAAATTTACTTGTTTTTATTCTGTTTTACATCAATCGACTTGGCTTCAGCTCTTACT

GCCCCCAAAGAACAACAATTGGCATGTTTAAATGAGAACGGAGTAACTAATTCTACTGAC

GTGGAACTCTTTAACATAATTATGCAAGATGTTTTTAAGGATGAATTTATTCTGGAAACG

TCCCAGGATAAGCAGTTTAGCTGCGTTTTTGCTTATATAATTAATTCAATAATAAAGCGT

ATACCTCAAGATAGCATTTATCAGGAACTTACGCGTGTCATTCAAACTGTTATTATTTCC

ATGAAGAACAACTTTTATGGATTTTTTAGCAATGGAAGGTATATCATAATCGATAAATTG

AAATTTTCCTATAGA

>NvitOBP34(EMBL: HE578219)

ATGGGCTGTATGTGTAGTGTGATAAATGCTAATTTTCATTTTCGACGAGCTGCTGACGAC

AAATTAAGCGACGCGAAGAAGAGGGAGATGAAGGATACGCTTAAAGCTTGCAGTCTTGCC

ACTGGTGACGATGACGATTGCACTTTACTCCAGTGTGTTAGTATCTTGCAGCCACCTTTC

GTCTGGATACTTCCGAAGACGTCTGGAATTTTGGGGTATCTAAAAAATGTA

>NvitOBP35(EMBL: HE578220)

ATGAAATTGTTTTTCGCTCTCTTCGTCCTTTCCTTTGCGCTGCTGCATTCGGCAACTGGA

GCAAAAGACAGCTTGGTAGAATGTCTGCAAGAAAATGGACTCAAGATGGTTGACTTGGAT

TTTATGCGCAAAATCAAGCCAAACACCGACATGCCCAGGAATAAACTCATCGAGGATAAA

TTGGCTTGTGCTTTTGCCTGTTCCTTCAACAGGGATAACAGTTGGAAGGACGAGAATGTC

TTCACTTTCATGACTGACGTCATAAAAAAGGACTATCGCATACCAGTCGGCTTGAAAAAA

CAAATGCTCGACACGTTGAAGAGCTGCAATGCCGAAGCTAAGGGCGACGACTGCACATTG

CTGCAATGCATCAAGGTTACCAGGTATCCATTCATGGATTTTGTGTTTTTGCACAATACC

GAGATGAAGCCTGAATACGAAAGCAGTCAAGATAAACAAATA

>NvitOBP36(EMBL: HE578221)

ATGAAATTCGCGCTCCCCGCTCTTTTTATTATTTCTTGCGCAGCCATACATTTGACTGCT

GCAGCGAATACGGGAAACGACAGTATGACAGAGTGTTTGCAAAAATACGGACTCAAGATG

GATGACTTGGAATTCTTACGTAAGCCAAATACTGAGGTGCAGCCGATGAAAAATAAAGCT

ATAGAAGACAAAGTAGCTTGTGCCTTAGCTTGTACGTTTAAAAAAGAAAGTCAATATCGC

AGTATCTTTCCGTTTCTGAAAAACGTCTTGAGAATAGACAGACAAATACCAGTAAATTTG

AAAAAAGACATGCTTGATACGTTGGATGATTGCAATGGTGAAGCCAAGGGAAACGACTGT

AAGTTGTTGCAATGCATCAAAATTACTAGAAATCCATTCATGAATTTGGTGTTTTCGTAT

GGTGCT

>NvitOBP37(EMBL: HE578222)

ATGCAAAGTTACTTATTTTCCTTGATCGTCTGCTTTGCGTCAATGAACTTGTGTTTGGCC

TACTATTATACCGAAGAACAGCAACGGGAGTGTTTATTTAAAAATGGGTTAAATAATTCT

ACCGATATTGAACTGTTCAAAGAATTCATACGCACTGATTCCAAGGATCAAAAAGTATTT

CCCTTGGAAGACCAGTTCAGCTGCGCCGTTGCTTGTGTCTTTAATTTAGGAAAGCCTGAT

CCATCCGAGGATAGAATTTATCATAAACTTCTGTATTCCATTAAAAACGATGACCAAATC

CCAGGTGAACTCAAGAGATACATGATGGATAAATTGGATCATTGTCACCGGCAAGACGAA

GGTGACGACTGCAAGTTATTCATCTGTATCAAATTATTCAGACCTCCATTCAAGGAAACT

ATTATATCGATTGAA

>NvitOBP38(EMBL: HE578223)

ATGAAAATATTCGTGACCTTCTTTATTCTCTGCTTCGTTTGCAAACCGAGCAATGGAACT

TCCCTCGTGAAAGCCTGTCTCGTTGAAAATGGATTCGGAGCTAGTGGCAAGGACTTGGAA

ATCGTCAAAGCCGTGGCAAATCCCGAATACGTCGGTATATTGAGGCAAGTGCCAAAGGAC

AAGCTGTCTGGAGTTTTCGCCTGCATCTTTCAGGATAGGAATCCCACGACTAATCTCTAT

GCTTCCCTCAAACGCCTCATAGAAATGGATGATAAAGTGTCGAAGGCTGAAGCGCAAAAA

ATGCAAGACAGCCTGACTGATTGTCATAAAGAAGCTGGGGATGATGCTAAGTTACTGAAT

TGTGTGAACGCTTTTGACTCTCCATTCGATGAAATTATGGCTACTATTCGCGATGTGCCG

GATTCCCTGGGCTTTTGCTATGAAAAATGCAAGCTGACGATCAGTGAAATGTATAAGATC

GAGCAGAACCGATTGAATTTGAAGGAGATTATGACGTTCGTACCTGAAGAAAAGATGGCT

TGTACTACGGGCTGCAAAGTTGACAAGTCCGGCAGAAGCCTTACGAGCAGACTTACTGAC

TTGATTCACAAGAGTAAAAAACACGACGAAAAGAAGAAGAAGGAAATGATAGAGACTCTG

AATAGGTGTAGCGCGCAAGTTTCCGGAGTCAAGGTTGAAACCTACAATTTGATCAAATGT

CTGAATCTGTACAAGCCTCCCTTCATCGACCTTTAT

>NvitOBP39(EMBL: HE578224)

ATGAAGACGTTTACGATCATTTTGATTCTTGGCAAGGCTTATTGTCAATTTGGCAATGCC

TCTAGACTCGAGGACATGAAAACGTGCTTAGTCGAAAATGGTTTCACTGACAGTCATGCT

GACTTGGAGCGTCTACAGGCTATTGGAGATCCAGAACACGTCGATAGATTGAAGGACGTT

TCGAGAGAAAAAATGGCTGGCGTTATTGCTTGCATGTTCCAGAGAAGACACGGAAACAAA

AACTTAAACGAAGCCCTCCAAAATCTCGTAGAAAGAGATGATAAGGTGACAGAGGAGGAA

AGGAAAAAGATGTTGGAAACGTTGAAAAGTTGCAACGCAAATGCTACTGGAGATAATATT

AAACTGCTAAGCTGTCTTAACATTATGGCTCCTCCGTTTGATGTTCTGATAGCTGCTTTT

CGCGATCTTGATGATTCCGTCGGTATTTGTTTTCCTAGGTGCAATGTCACCATTGGTGAA

ATGTACAAAATGCAAAAGAACATGGAAATGAAAACCAAGGTTAAGGATCTGCTGAAAATT

GTCAACGAACAGAAAATGGGCTGTTTCATGACATGCATAGTTGATGAGGAGGCGAAAATT

AATCGACTCAGCCCGCCTTTTTTAAGGGTGCTTACGGACCGTATTAATAAGAGTGAAAAT

CACGATGAAGATCAGAAGAGAGAAATGCTGAAAACTTTGAGTAAATGCAACGCACAAGTT

GCTGAAGTTAAGGACAAAACTTATGAAATGATCAAATGCGTCAATATGTTCAAGCCTCCA

TTTATCGATATTTAT

>NvitOBP40(EMBL: HE578225)

ATGAAGACATTTGCAATTATTTTGATTCTCGGCTTCGCTTGGCGAGTGAGCAATGCCTCT

AAATATGAGAACATGAAAACGTGCTTAGTCGAAAATGGATTTACGGACGATGAAACTGAC

TTAGAGCTCATACGGGCCATCGGAGAGCCGGAACATGTCGATAGATTGCAGGACGTTTCG

ATGGAAAAAATGGCTGGCGTTATGGCTTGCCTGTTTGAAAAACAACAAGGAAATGGAAAT

TTAAATAATGCCCTTGAAAGTCTTGTAGGAAGAGATGATAAGGCGACAGAAGAAGAAAAG

AGAAAGATGCTGGAAACGCTGAAAACTTGCAACACAAATGCTGCCGGAGATAATACTAAA

CTGCTAAGCTGTCTTAACATTATGGCTCCTCCGTTTGACGTTCTCATAGCTAGTATTCGT

GATTTTGATGAATCTGTTGCTGTTTGTTTCCCTAAGTGCGAAATTACGATTGGGGAAATG

TACAAAATGGAAGAGAACAAAAGTAAGGTGAAAGGGCTGTTGGAAATTGTTAACGAACAG

AAATTGGCTTGCTTTATGGCATGCATAGTCGAAGAGGAGGAAAAGAATCGAAAAAGTCCA

CATTTTTTGAAGGCACTGACGGACCTTATTAATAAAAGCGAAGAACATGACGAAAATCAG

AAGAAAGAAATGTTGGAAACTGTTGATAAGTGCAATGCACAAGTTGCAGAAGTTAAAGAC

AAAACTTACCGAATAATCAAATGCGTCAACATGTTCAAGCCTCCATTTGTCGATCTTTAT

>NvitOBP41(EMBL: HE578226)

ATGAAGACGTTCGTTTTCATTTTGCTTATCGTCTATGTTTGTCAGTGGAGTGACGCTCGT

GAAACCGATTTCGAAGATCTTATATTCAATGACCTTATGTCGATGTGTTTAATTGAAAAT

GGATTTACTAATTCCACTGCTGACGTGGAGCGAATGTATGCCATCGGAGATCCTGAACAA

GCCGATAAATTGAAGGATGTACCGAAAGAAAAAATTGTCGATGTTATCGTTTGTCTATAT

CATAAACTATACCCATACCGAAATTTGTATCCTAGCCTTAGGCAGCTCATCGACAGAGAT

GATACGGTAACTGTGGGTCAAATCAGACAAATGGAACACACGATGTCTGATTGCTATAAG

GAAGTTGGTGGAAAGATTAATAATGCCGAACTTCTAAAGTGCGTAGACATTACCGAGCCT

CCCTTCGATCATCTTTTCGCTGCTATTCGAGATGTCCGTCAAGCTTTGCAGTGGTGCTTC

GTAAGATGCGGAGTATTGATTAGTGAAATGTATACGATGGAAAAAAACCGGGACTTGCCC

TTCAAGGAATACATAAAATATATTCCTCATGAAAGAATAAGCTGTTTGATGGCTTGCAAA

GCTGAGCAAGCCGTCGTTAATAATTCTGATCAAACTCTTCAAAAGGGACTTGCCGACCTG

ATCAAGAGGAGTAAAAATGTTGACGAGATAGAAAAGGCAGAAATGTTGAAATCATTGAAG

AAATGTAGCAAAAAAGTTTATGGTTATGAAGATGAACATTACGAACTAGTCAAATGCATT

GATCTGTTTAAACCACCTTTCATAGATCTTTTT

>NvitOBP42(EMBL: HE578227)

ATGCATTATGCAATCTTCTATATCTGTATAACGTTCGCTATCATTTTGGTTCTTGCTTTT

GTTTGGCAACCAATCACTGCCAGCTTCCGTGTCAAATACTCGGAGACATGTTTGATGGAA

AATGGATTTACCGACAGTCCTTCCGTTGTTGCCTGTGTCTACCAAAAACAATACGCCGAC

AAAAATTTAAACAATGCCCTCAAAGTTCTAATAGACAGGGATGATAAGGTATCGGAGGAT

GAAGCGAAAAAAATGAAGGAAACGTTGGCTACTTGCAACACAAATGCTGCTGGAGATAAC

GCTAAATTGCTGAGCTGTGTAAACTTAGTCGCTCCTCCTTTCGATACTCTCATAGCTGTT

ATTCGTGATTTACCCGAATCTCAGGATCCATGCTTCTTGAAATGCGACATGAAAATCGGT

ATGCCGATGAATATACAAATAATAACAATATCCTCAGTTATCACAGAAAATGGCATACGA

TTATCTGATTCGCATTTTTCAGGTGAAATGTACAAAGCGGAGCAGAATCGAAATAAAATG

AAAGATCTTGTGAAGCATGTCCCCGAAGAAAAACTAAGCTGTATATTTGGTTGTAAAGTG

GACGAGTTCGATAAAACGAAACCGGGCGGTAAAGTACACGACGAGAACCATAAAAAAGAA

ATGTTAGAAACTTTGAAGAGGTGCACCGACACAGTTGCTGGACAACCGAATGAAAACACT

CTGTTAGGGAAATGTCTTGATCTGTTTAATCCTCCATTCGTCGATATTTAT

>NvitOBP43(EMBL: HE578228)

ATGAAGACGTTTGCGGTCGTTTTAGTTATTGGGTTCGCTTTGCAAGTGAGCAATGCGTTA

ATGCCCAGTGATCTGGAAACGGTAGTGGAAACGTGTCTAGTCGAGAATGAATTTACAAGT

AGTAAAGATGATGTGGCTCTCGTCGATTACATCGGAAAGCCAGAAGGTGTTGATAAACTA

AAAGATGTCCCGAGAGAAAAGCTAGCTGCGGTTTTGGCCTGCATGTACCATAAGCAGTAT

AAAAATACAACTTTGTACGACATGCTTAAATATCTTACAGAAATGGATGATAAGGTAACT

AAGGACCAGTACCAACAGATGAAGGAAACCTTGACTACTTGTGTTCAACAATGTAATGGA

ATAAATTTAATACGAAGAGATTACCCTCAAGGAAATGACGTAGAATTACTAAAATGCGTG

AAGGCTGTTGATCCTCCTTTCGACTATCTCATAGCTACTCTTCGCGACCTTGGCGAGGAA

CCTCTGAATTTTTGCTACGAAAGATGCGGACTAAAGATTCTCATAAGAATTTTGATTATC

AGGAGCTTACGTACATTGCACACGCGGGATTAT

>NvitOBP44(EMBL: HE578229)

ATGAAACTACATTTTTACGCGATGAACACGTTAGTTTCTTTTTTCGTTCTCTGCTTTGTT

TGGCAATTGAGTCACGCGATTGCATTTTCGGATCTGACAACGTGCTTGATTGAAAATGGA

TTTACCAATACCACTTCTGATCTGAAACGAATATATGCCATAGGAGATCCCAAACAAGCT

GATAAACTTAAAGATGTTTCGAAGGAAAAAATGGTTAATGTTGTAGCTTGTCTGTACGAC

AAGCTGTACCCACACCAGAAGTTGTATCCAAGCCTTAGGCAGCTTATTGACAGAGATGAC

ACGGTAACTTCGGATCAAATCGAGCAAATGGAAAATACAGCGTCTGCTTGCTATAGGAAA

GTTGATGGAAAGATTAATAATGTTAAATTTCTGAACTGTGTAAACATTACCGAAGCCCCA

TTTGATCATCTCTTCGCTACTATTCGAGATGTCCAACAAGCTTTGCAGTGGTGCTATGTA

AGATGCAGAATATTGATTAGTGAAATGTATACATTGCAGCGAAACCGCTACAATCCTCTG

AAGGATTACATACAATATATTCCTACTGATAAATTAGGTTGTTTCATGGCTTGCAAAGCT

GAGGAAAGCACAATGAAGAATCCTGACCAAACTCTTCAGAAGAGGCTTACAGATCTGATA

AATTTGAAAAAAAATATTGACGAGAGTAAAAGGAAAGAAATGTTGCAGACTTTGAACAAA

TGTCTTACGCAAGTTCCTGCCGACGAAGACGAACGCTATTACGAAGTAATCAAATGTATC

AATCTATTCCAGCCACCATTCGTTGATATTCTTTACAATAACTTTTATAGCGCTTATAAC

ATAGAAAAAAAAGAACTGAACATC

>NvitOBP45(EMBL: HE578230)

ATGAAGACGTTCGCGATCATTTTATGTCTCGGCTTCACGTCGCAAGTGAGTCACGCATTC

TTCCCCCCTGACGTGGAAAAGTGCATGGTCGAGAGTGGATTTACGAATATTCCAGATGAT

TTGGGAATCATAGACGGCGTAGGTAATCCAAACGATACCGATAAACTGAAAGATGTTCCG

AAAGAAAAATTGGCAATCGCCACGGCTTGCATGTTCCACAAAGAGTATACGAATACTCTG

TACGATATGCTCAAGTTACTTATAGGCATGAACGATAAGGCAACCAATGATCAACGTCAA

CAAATGATGGATACCTTAAACACTTGTCATTCTGAAGTTAAGGATAACGACGCTGAATTA

CTGATGTGTGTGAAAGCTTTCGAGGCTCCTTTCGACTACTTCATAGAAACTCTCCACATG

TTTGGGAAATCGGCTCTTTATCCTTGCTACGGACACTGTGGATTAAAGATTCCCGACTTG

TACATTTTGGAGGAGCATCGCAATCACGTTGACGAGCTTCTCAAGCGTATCGGCACAGAC

AACGTGTACTGCATTACAGCTTGTAAGGTAGAACGTAAGGAGGATAATCCAGCACTGGCT

CTCTACACCATGCTTTCGGAAATCATCGATAGAAGACTGAAGGACGACAAGAATACAGAG

GCGAAAGCCGCGCTGGAGAAGTGCTATACCGACGGTGAATACCAAAAAGTTGAAATGGCA

GGCAGTTTGGGCGAGACTATTCCCAAAGGGTCCAAAGTGCGAGATACTTACCAACTGTTG

AAATGCATGAAAGTGTTCGAGGAACCATGGATTAATCTTATGAGAAAT

>NvitOBP46(EMBL: HE578231)

ATGAAACGATTTCTGATCCTCTTCATTTTTTGCCTCACGTCGCCACTGGGACGAGCCGAC

ATTTACGACGACACCGTGACATGCTTGAAAGAACGTGGGCTCAAGGAGACCGACATAAGG

ATACTGGACGCCGTCGGTGAGCCTGGAAACGAGCACATACTGAAAGATGCCCCGAAGGAC

AAACTGGTCGACGCCATTGCCTGCATCTTCCGCAAGGATCACAAAACCAACAAGGCCCTC

TTCGGCACCCTCACGGAGCTCATCAACATGGATAGCGAAATCGACAACAACAAGAGGAAG

GAGCTGCTGGTGACCTTGAACGCTTGCAATAAAAAAACTGATGGAAATGACACCAATCTC

TTGAAATGCCTCGAAGCTACTCAGCCTCCATTCGACAAATACGCCGCTATAATTCGCGAT

GTGACGAACGTCATCAAAGAGTGCTTCTTCTCGAACAAGCTGACATTCAGTGACCTACGC

TACAGAAAGATGCTGCACAGGGAGTCGGTGTCGGAGGTGCTGAAACACGTCGGCGAGGAC

AACATCACCTGCGCCATGACCTGCGAATTCGACAAGAATAAGCACGTTTACAAGGACATG

GATCCGAACGAGACTATTCACAAGAGGCTCGAGACGCTGATCACTAAGAGCAGCCTACCG

GAGGAGAAGAAGAGGGAAATGAAGGAGACCCTGGACCGATGCGATCACCAAGCCCGAGGA

GACTCTTGCAAGTTCGGAAAGTGCGTCAAGCTGATGAGGCCGCCGTTCGTGCAGCTGTAT

TTTAAAAACCCC

>NvitOBP47(EMBL: HE578232)

ATGAAAAGCTTCTTGATTATCCAACTGATCTGCCTCTCCGGACTATTGCGCGCGGTGAAC

GCGGAGAGCGTGACGACGTGCCTGGTAAAGAATGGGATAAAATCCATCGACACGGCGACT

TTCGAGGAGGTGGTCAAGACGAACGGCGACGCTTTGAAAACCGTGGATAAGCAAGTCGCC

TGCGTGATCGCCTGTGCTTTCGACGACTTCACGCAGAAGAAAGAGTCGATCTACAATTAC

CTCACGAGGATAATAAAGAAAGATCTGGACGACGAGTACTCGGCGAGCGAACGCAAAGAG

ATGCTGGATATTTTGAATTACTGCAATGGTCTAGCTTTCGGCAACGACTGCAAATTTCTG

CATTGTATCAATGTGACCAAGGCCCCGTTCGCCGATTTGATTTTAACAGTCGGCATCGAG

GACAGTGGAAAGGGCGAAGATCAGAAAATGTTTATG

>NvitOBP48(EMBL: HE578233)

ATGAAGAACTTATCCATCGTATTGATTGCCTGCTTCGCAGTTTTCCAGTTGACTAGTGCT

GACTCAAATCACATTAAGGAATGCTTGACAGAACATGGACTCCAAGAGACTGATATGAAT

TTACTTAAATCCCTCGGCAGCGTAAAATTTGCGGATACTACAATAAAGGACGAAGACAGA

GAAAAATTAGACTGCGTTTTAGCATGCATTTTCCAAAAGGAAAGTCCTGGACAAAGCCTT

CACGAAGTGATCAAAGATGTCCTGTCCGAAGATACCTACAAGTCGGAAAATCTGCGCAAA

GAGATGACGGAGACCCTGAACACTTGTGAAACTCAAGCCGGTGATGATGACTGTAAACTG

CTTCAGTGTGTCCAAATAACCAAGCTTCCATTCTACGATCTCCTGTATACTTTTGTTGTC

GCCAGGGACATGCAAAAGTGTTTCGTCGACAACGGACTTAATGCTTCTGACGCAGTAAAC

TTGGAACACTTAAACGAGCCAGGATGGGTGGAGGAGATTTTGAAAAAGGTGGACGAGGAC

AAACTGGCGTGTACAATGTTCTGCCTTTATTCCAAGCAAGATAAAATAACACACGATAAA

TCACTACAACAGAGATTCGAAACTAGGATCAATGAGAGCGACAAGCTTATAGCTGATCAG

AAGAAGGAAATGCTCGAAACGCTGAACAGATGCGGTATCGAAGCGGCAGGAGACAACTGC

AAATTGGTGAAATGCATCAAAATATTAAGGGCACCGTTCTTCAATCTGTATCATCAA

>NvitOBP49(EMBL: HE578234)

ATGAAACGTTTTATAATAATAGTCTGCTTTTACTTTATATATTTACACACGAGTAGCGCT

AGGCGTTCCGACAACTTGAAGAAATGTTTGGTCGAGCATGGATTAAGAAATAAATTATAT

AAAAGAGCAATCGGTGGTTCAAACATAAACAGTTTGGATCGAGATGTGAATGTTGATTTG

AAAGAAAAATTGGCTTGCACTTTGGCGTGTTCCTTTCGCAAAGAGCATTCAGGCAACGAA

ACTCTTCATGCAATTTTCACAGAAGCTTTGAATTTTGGAAATTTTCACATTTTGGAAGAT

GTAAAACAACAAATGCGAGAAACTTTGAATAACTGTTACACTGAAGTTGAGAGCAATGAC

TGCAAGTTACTTCACTGCATTAAAATAATTGAGTCACAATTTATGGACGTTGTTCTCGCA

ATAACTAATGAAAGACCAGTGAGATTAAGCTTCGAAAATACCAATTCCGTCAAAAAATTT

AAAAAGATAAAATTTCGCATCAAGTGGTTCAAAGTGATGCATATGATGATAAGATCGCTA

CAGCGGCATATTCCATACAAACAA

>NvitOBP50(EMBL: HE578235)

ATGAAACGTTCCCTGGTCGTTTTGTCCTTTTGCTTCCTACTGTTGCAATCTGTGAGGATC

GTGGCTGACGATGAATCGGAGGAACTGAAAGAATGTTTGCAGGATAATGGACTTCCAACA

AATATCCATAAGGAGGTGGGAAAGGGCGAATTTGAGAAGTTGAAAGACGTTCCGGAAAGC

AAAATTGCCTGCGTAATAGCATGTTCGTTCAACAAACGTATACGAAGCAACGATTTTCTT

TTCACTTCCTTTGTAAAAGCTGTGAATAAAGATAAATCATTAGGAGAGGACCAGAAAAAA

GAAATGCTAGAAACCATTAATAGATGCAGCAAAGAAGCCAAGGGTGACAACTGTAAATTT

TTAGACTGTCTTAAGATAACGAAACCGCCATTCGTAAACTTAATCATTACACCGAAAGAT

GCGAATGATAAAAAAATT

>NvitOBP51(EMBL: HE578236)

ATGAATAGATTGTTCATCTTCTTGATTGTACTCGTAGCAGGATCACGTGCGGACAAAAGA

CATGATTATGGACAGAAAATGTCGCAGTGTTTGATCGAAAATGGCCTCAACGAAACTCAT

CTAAAGTACGTGTTTAGAATAGGAAAGGTAAATTTGGAAATACCCAGTGATGTTTCTGAA

GAGGCATTGGCTTGCATGTTAGCGTGTGTCTATAACTTTACCATCACCATGAGGGAAACT

CAAAAAACGTTGGATGAAGCTATTCTGGATGTCATTAATCTAGACGATATGTTCAACAAC

GATGAAAATAAACGTAAAGCTTTGAAGGAAACTCTAGATAAATGCAAAACAGAAGCTGCA

GGGGACAATTGCAAATTGCTGCAATGCATCAAGGTTACCAGAGATCCATTCGACAAAATC

ATTACAGCTGGTAGATACAACTACGGTTACTTGAGAAGAGATAATGAG

>NvitOBP52(EMBL: HE578237)

ATGAAAAATCTCTTTGTTATACTGGCGCTATGTGTCGTAAGCTTGTATGCGCTGGATGGC

TTCGATTTCTATCGAAAGAAAATGGATGAATGTCTATCCGAGAACGGGCTGAGCGAATAC

GATCTGAAGTACGTGTTCAAAATAGGAAAACCAGATTATGAAAGTCCAAAGAACATATCC

GACAAAACGTTGGCCTGTATGCTTTCTTGCATGTACCATCTGTACAAGAATCGTCAAACT

ATCGATAAGGCCATTGCAACCGTGATAAATAGAGACGATAGTCTCACTGCTGAGAAGAAA

AAGAATTTGCTGGGAACTGTGCGGAATTGCACTCGAGAAGCTGGCGATGATGATTGTGTA

CTGTTGCATTGTCTCGATGTAACTAAGGAACCGTTTTCAAAAATCATAACAGCTGGTAAG

AAGATTAAACCTAGCAAGATTGACACAGAC

>NvitOBP53(EMBL: HE578238)

ATGAAAATACTGCTATTCCTCTTGATTTTCTGTGTGGTTGGAATTTACACGCAAAAACAT

AATGACTCCGCTGAAAAAAATAAACATGCCATGGATATAGAAGATTGCTTGAATCAACAT

AGTAACATAACGAAAAAAGGTTTATCTGTAAAAGATATCATATTAAAATCTATTGCCCCC

TACGATTTGGGCTGCATTACATCATGTTTAAAGAAGAAAGAATTGAAGAATGGAGTAACT

TTAAACAGTTACGTTATACAAAACGCATATCTACCGTCTACGAAATTCCCTGATTGGTAT

GAAAAGAAAAACGAAGACTATCAGTACGTTGTAATAGCAAATAGATGCATAAACGAAGCT

AAAGAAGATGAATGTAAACTTTTCATGTGTCTGAAAGCTTGGGAACTTCCATTTGCGCAT

ATTCTGTCAATAAAAATCGAACGCTACATTACATTC

>NvitOBP54(EMBL: HE578239)

ATGAAGCTTTGCTGGATTATTCTGATAGCTTTATGCATTTTTGGAATTAATGCTCGACCC

AACTCAGAACCGGATAACGATGGCGGATTCGAGCCCTTGGCTCTTCAATGCTTGAGAGAA

TTGAAAAAAGATCCAACTCTGTCTGCGAAGAACTGCGACGAAATAGAGAGCAGCTTGACC

GACGATGAGAGAAATTGCATCCTAGCTTGCATGTTCAGAAGAAACGATCCCGACAAGAAA

AGCTTGTACGAATACTTGAAGAGTCAACTAAGCACAATCGACAATCGAATCCAAGTGTAC

CGTGATGAGTTACTTGAAAAGCTAAACTCGTGCAAAGCTTTAGTTGGAGAAGGCAACGAC

TGCGGAGTTATGAAATGTATAGAGCTTTTCAAGCCGCCATTTGCTCATTGGTACTTGCAA

ACGAAT

>NvitOBP55(EMBL: HE578240)

ATGAGGAACTCTCTCGTCGTCATCGTGCTCATTTGCTTCTCTCAAATACACGCGATACCA

CTAACCAAAAAAAAAGAAAAATTCGTGCCAGCCGAGGACTCTAAGGAACAGTGCATGATA

AAATTCGGGCTCGATCCAGATTTCGTGGATTACCTGATCGGACTGCACAGACCGCAAATC

GAAATCAATGCCTATATCGGAAGTAAGCACTCGTGCATTCACGCTTGTATGGTGAAATTG

GATCAAAATTTGAACCCGTACGATTACGTCGTCGATCGCGTATCCGCCGATACCAAAGAG

GAATACGAGCGATTGATAAAGCTGGTCAATAAATGCAACGAGAAAGACTCCGGAAACGGT

TGCGTATTACTGGACTGTGTCAGACGGAACAAGGAGCTGAGAGATTTCGTTTAT

>NvitOBP56(EMBL: HE578241)

ATGAAGCTTTTCGTATTTTGTGTTTTTGCGCTTTGTTTAACTGCCGCTAACGCTCTATTC

GGGCCAAAACTGAAAGAAAAATTGCTAGAGAGGGAAGATGCCTGTCTCCGGGAAACAGGA

AATACGTTACTCAGCATCGATCATGTCAGACGCACAAAAACACTGCCCGAAGACGGTAGC

CTCGACAAATTTGCACTTTGCTTATTGAAAAAACATCGCATAGTCAACGACGACGACACG

GTGAACAAAGATAAACATCGATACTACTTAATTTTGGACGACGGCCGTAAGAAGGAGTAT

GCCGAGGATTGCGTGCTCAGCAGCGGCGGTTCGAACAACGGCGAAATCGCGAGGCACCTG

CTGTCCTGTTTGCTGAAAACCGACATATTCTTCATCGACTGGTCGTACAGATCCCAAGAG

GTTCTCTCGCAAATGCGTCAGAGGCAGAAGCAAAAGACACAAGCA

>NvitOBP57(EMBL: HE578242)

TTGACATTTAATTTCCGAACAAACACTGACATATTGTTTCAAGCTTCAACTGGTAACATC

GTCAATGACACTTTAAACAGAAAATTTTTGCTTCTTGTCAAAACATGCGCAAATAAAAAA

CTGCACATGTCGGACTATGGATCAATAAACGAAGACGTCAACTCGTGCCATCTCATCTTC

GATAATTCGTCTATGCTCGAAACCGTCGAAAAGTGTAAAAACCGAAGAGAGACTGCCAGC

AGAGACATGACCTGCTTCTTAAAGTCGCACGTGCTCATCATTGATCCTTACATTGGAGCA

CATGAAAGCGCC

>NvitOBP58(EMBL: HE578243)

ATGAAGTTTTTCATCAGCTGTGTTCTTGTTATCTTCTGCTCGTCTTCTGCTATTGGTTTG

CTTTCTCACGAAGCCATCCTGAGCCTACAAAGGGACCAGGACGATTGCGTCAGGGAAAGC

GGTGTTACTCGGAGTACCGTCGAACAAGCCCATCTCGATCGAGTGATACACAACGACGAG

AATATGGCGAAATTCGCAGCCTGTATGCTGAAGAAATTCAACGTCATGTCTGACGACGGA

AAGATAAACGAAGACGTCTACTCGTACCATCTCATCTCCGACAATCCAGCGATGTTCGAA

ACCGCCGAAAAGTGTAAAAAGCGAACCGGCAGTGACGTGGACGAGACGGCCAGCAAAATC

ATGACCTGCTTCCTAAACTCGGACGTGTTCGTCCTTGATCCTTACATCGGAGTGCATAAA

CGAGCT

>NvitOBP59(EMBL: HE578244)

ATGAAATTTTACGCGCTTTGCGTTATCTTGCTGTGTTCGTCGGCTGCTTTCGCCTTGCTT

GAAGCGAGAGTCCGCGATTATTTATACGAATACCAACGTGACTGCATGATAGAAAGTGGA

GCTGATACTTCCCTCGTTGCAGCTGCTGATAGAGCTAGGATAATTCCCAATGACGGGTTG

TTAGACACATTCGCAATTTGTATGCTCAAGAAGTACAATATTTTGCATAAGGATGGCTCA

GTCAACCAAGATCATGACTCGTACACAATATTCAGCGACAATCCGGATGTATACCGAATA

TCCGAAAGATGCAAGGCCAAGATTGGTAAAGACGCTGGTGAAACCGCGAGAAAAATTATG

AATTGTTTTGCCGAAGATGGGGACTCGTTGCTGCCTTACAGTACTCATCCGCCTCCCACC

CCCTGC

>NvitOBP60(EMBL: HE578245)

ATGAAAATATACGTTCTTTGTGCTGTACTTTTCTTCACACCCACTGTATTTGGTATCTAT

TCCTCTGCAATTTGGGATGCTTTACTTCATGCTAACGAAGAACCATGTGGACGAAGTGCA

GGACTAAGCGAAGAAAGTATCGAAAGTTCCAGACGAGCCAGGTATCTTCCCGAAAGTCCA

GAAATGAATGTATTCGCATTTTGTGTAATACGAGTATTAAATATCATGAGCAAAGATGGA

AAGGTAAATCCCGACATTGGATCATACCTTGTTCCGACTAACACACCGGATATTACAAAG

GTAATTTCCGAGAAATGCAGAACTCATGTTGGAGTTGATGCCGGAGATACCGCGAGGACA

ATTTTGAACTGTTATCTCCAAGCAGATCAACTGGTCATTTCGTTACCCTCAGACGCGCAA

CTGACTTTTAAC

>NvitOBP61(EMBL: HE578246)

ATGAAAATATACGTGATTTGTGCTGTGCTTTTGTTCGCACCAGCAGCATTGGGTCTGTTT

TCTAATGGAATTTGGGATGTTTTACATGCTAATGAAGCAAAGTGTCAACTCAATAGCGGA

GCAAGCGATGCAAGTATCGAAGATGCCAGACGAGCTAGGAAACTTTCCGAAAGTCCAGAA

ATGAATGCCTTCGCGAAGTGTATGCTGGGAATATACAATGTTATGAGACCTGATGGAAGC

ATCAATCCCGATTTTCAGTCATACACTGTACCGACCGATGTACCCAATAATACCTGGAGA

ATTTCTCAGAAATGCATAACTCTGGGTGGAACTGATTCCGGAGATACTGCGAGAAAAATC

TTCAACTGTTATACTGAGAACAATCAACTGGTTATGGCCTGGACACCTAAAGTGTCTGTT

>NvitOBP62(EMBL: HE578247)

ATGAAAATATACGTGATTTGCGTCGTTGTTTTTTTCCTTGCACCAGCTGTTTTTGCTAGC

TTTTCTCCGTTAATAGAAGATGATTTTCACGCTTATGAAGCAGATTGTGGAGCGAGTGAT

GAGAGCATCGAAGCTGCTAGACGAGCCAGGCAACTACCCCAAAGCCCACAAATGAACGCG

TTCGCTTTGTGCATGATGCAAAAATACAAAGTCATGGCTGCAGATGGATCCGTTAATCCC

GACGTTCGGTCTTACGGAATCATCACTGACGGCCCTGACAATACCTGGAGAGTTTCTGAA

CATTGTAGAACTTTAAATGGAAACAGCGCCGGAGAAACCGCGAGGATGATAATGAACTGT

TACCTGGACAATAATCAGTTGGTGATGGGCTTGACTCCTCGAGTTTCCGCT

>NvitOBP63(EMBL: HE578248)

GGAACTTTTCTACCCTCAATAGATGATACATTGCATGAATATGAAACGAACTGCGCTCGG

GTCAGCGGCGCTACACATTCTGCTATCGAAATAGCCAGAAACACCAAAATGTTGGCTAAT

ACAGCTCGACTAAATGCCTTCGCAATGTGTATGCTTCAACAATTCAATGTGATGGACAGC

AATGGAATTGTGAATCCCGACGTTATGTCCTACAGTATCATCTCTAATGTGCCAAATGCC

ACTGCTGGCATTTCTCAACAATGTATTTCCAAAAGAGGCATAGACGCTGTGAACACTGCG

AGAATGATCATGAACTGCTATCTGAGAGCTAATCAAATGGTTTTGGCGCTTTCTCGTCGA

GATTGTACT

>NvitOBP64(EMBL: HE578249)

ATGAAGAAGTTTACGCTGATTTTTGTCTCGTGCTATCTCGTTTTTTCATCGATGCATCGA

GTTATGTGCGTCACCCAGTGCTTTTTCAATGAACTCAATCTGGTTGATCAAAGAGGCTTT

CCGGAAAGATCGGCCGTAATTGGCATAATGACTCAGAACATACAAGATCCAGAACTGCGA

GATTTCGTGGAGGAATCTGTGATCGAGTGTTATCACTACATTAACAACAATAACAGCGGC

CGTCAAGAGAAATGTCAGTTTTCGCAAAGTTTGCTGTCCTGTCTGGCAGAGAAAGGCAGC

GAGAGATGCGAGGACTGGGATGACGAG

>NvitOBP65(EMBL: HE578250)

ATGAAAAGCATACTTTTCATCTTTGCTATCGTCTGTGTCGTTGGAGTCTTTTCGGACGAC

GACAAAAAGGATCTTACCCGTGAACAAATTCTAGAATGCGTGGCGGAGTCAGGAGTCGAT

GAGACGAAAGTAGAAGACATAAAGCTTGGCAATCAGGGATTAGAAACGACCCGGGAAATC

GATTGCTTTGCAGCATGTGTTTTCAAAAAACAAGGCATCATGAATGAGGCAGGTGTAATT

ACTCCGGATAAGCCAATGGACAATGAAGCAGCCAAACAATGTGTAGCTACGACTGGTGCT

GATGCCTGTGACACAGCCGGTAAAGTGCTCAAATGCTTCATTTCTAATAATTTGGTCTCC

CTCATGGACCTGGACGACGAC

>NvitOBP66(EMBL: HE578251)

ATGAAGTCTGTACTAGTTGTGTTTGCTGCAATCTGCATCGCCGGTGTGCTCTCGGACCCG

AAAGGAGATATCGATGCTTGCGTCGCTGAATCAAAAGTCGATACAAAATTGTTTGAAGAT

ATGATGCACACCCCTGACTTCAAGGCTACTCGAGAAATGGACTGCTTTGCTGCCTGCATG

TTCAAGAAAGACGGCGTGCTCGATGCTGATGGTAACGTCGACGCATCTAAACTACCAAAT

GTTGATGTCAGCAAAGTTTGCGGAGCACTTCGTGGGAAAGATGCATGTGAAACTGCCGGC

AAGATAATAGGATGTTTCGCCGAGAAGGGCGTCATGGATGTGTTTCATATAGTA

>NvitOBP67(EMBL: HE578252)

ATGAAAACTTCAGCACTATTATTAGTTGCTTTTGGAATCTTTGCTTTTACCGAATTATCC

ACTGCCTCATTAGATAAATGGTTTGAAGAGTGTGTTAAATCCTATGGTCATACTGAAGAA

AGTGTTTCAAAACTTCCCGACTTAGAAAAATCTTGTGTGATACACATCTGTTTCATGAGA

GATGTTGGACTTATCAACGAAGACAACAGTTTAAACGTGAACTATCTACTCGAACGAAGA

AAGTCTCACGTTCCTGAGTCCAAAATATACGATGCTGTTAGAACTTGCAATGCTGAGTCA

ATTGACACTCTTGCAAAAACATGTGAAGCAGTTAAGTGTTTGATGGATTTGTTACACGAA

TCGGACTTCAACACCCAACCTAACGTCACTGAT

>NvitOBP68(EMBL: HE578253)

CTTTTTGCTTTTGCAGTCTTCGCTTTTACCAATGTGCTAAATCCTATGTATTTTCACACT

TTTTATGAAACGACTTTCTTCTTATCTTGTGTAGAAAGTATTTTCAAACTGACCGAGTCT

GAAAGATCTTGTGCATTTCAAACCAGTTTCCTGAGAGAATTGGGTCTGATCAATAAAGAC

AACAGTTTCAATGTCAACGATCTTCTGAAACAAAGAAAGTCTGGCATTCCCGAGTCTAAA

ATACACGATGCTGTCAAAACTTGCGATGTTGAGTCGCTCGACTCTCTTGAAAAAACTAGC

AAAGCAGTTAAATGTTTGATGGGCTTGTTACGCAACATGTGGCTTATG

>NvitOBP69(EMBL: HE578254)

ATGAAGCTGTTCGCCGTTGTACTCGTGTTTTTCGCATTGGGATCGAGTTCGGTCGCTCTC

GATGAGGAGGAACGCGGAGTGCTGCGTCAGATACGTAACGTTTGCGTTGTCGAATCTGGC

TTGAGTCCATACGAGCTTGGATTCATCTACAGAGCCATTCGTCCAGCCAAAAAGTTGGCG

CAGGCTTCGAGATGCGTCATCCAGAAAATTAGCGAACTACAAAGCGAAAATGAGACAGTA

AAACACATCGCTGACCGCGGGAAGGCTGCCCTCGCGAACGCTCCTATTTCAAATATCGCG

GACAACGTCCTAGGCTCCTGTCAGAATTTGCTTGGACAAAATGGCTGCATACAGGTGTTG

GAACTCGCAGCCAAGATCATCGACAACCTCCGAAGCCGACGGCAA

>NvitOBP70(EMBL: HE578255)

ATGATATCTGGAGAAGAAATCTCCCTGCTAGTTATTTTCACTATCTGTTGGGGAATTAAC

TTAAAATGTAAGCATGCTGGGGAGATACAGCTTCATCTACAAGACAAAGAAGCGGCAGAA

AAATGCTCTAAAGACATTGGTATTACTCTTGAGACTGTATACGCTACTATGAAAAATGAA

TTGAAAGATGCAGACGAAAAACTCAAATGCTTTGCAGCTTGTGTTTTTAAAGAAAAAGAA

ATGTTAAAGGATGATGGACCAATCAACGTTGCAAAAGCAATCGAAGATTTACCCGATGAA

ATTAAAGATGATGTAAGAGATGCTATGATCAAGACAATCGAAAAATGCTCCCAAAAAAAG

GAAGCTAATGAATGTGAGACTGTTTTTCACGCTGTACAGTGCGCCACGCTAGATATGTCG

AAGTTGAAATTATTT

>NvitOBP71(EMBL: HE578256)

ATGAAGGTAGCCATAGTGGCTTGTGTTTTGACAATCTGCAGCATTTTTGCGGGAAGCAAG

GCGGACTTGACGGAAGACCAAAGGAAAATTCTCCAACCTTTAAAAGACGAATGTTTCCAA

GAAACTGGACTTGATGCAGTTACGTTGGAGAAATTTAAGAAGGAAGCTTTGCAAAAGTTC

AAGACGACTGGAGAAGTTAGTAATGATGAAAAAGTAAATTGTTTTTCGGCTTGCATGTTC

AAGAAAATTGGATTCATGTCCGAGGAAGGTAAATTTGAAGAAGATACAGTTCGTGCTTTG

ATGTCTGAAAATTTCCCTCCAGAGACCTTAGATAAAGCTATCGAAAACTGCAAAAATGAA

GTCGGAAAGGACCACTGTGAAACTGCGGCGAAACTCATAGTCTGCTTCATGAATAACAAA

GCTGGAATGGAGAACGTA

>NvitOBP72(EMBL: HE578257)

ATGTTATTTTTCACAGTCGTGCTTCTGTTCTCTAGCGTATGCACAGCTACAAAAGAGGAA

GAAGAATTTAAAAGCGAATTGGCTGAATGCAAGAATCTAGTTGGAGTCACTGAGGATTAT

GTAAGAGACGTTTTCAAAAGTGGATTAAAGGGTGCCGATGAAAAATTTAAATGTTTCATA

GCTTGTCTAATCCAAGATTCGTACAAGTTTAACGACGGTGGGGTATTCGATGCCGAAAGA

ACCATTGCAAATGATCGCGGTCCAGCTGGACTATTGCGCGATTACACAAACAAAGCTTTG

AAGGCATGCTCTAATATAAAAGGATATAGCGAGTGTGATGCTATTTTTAAAGTTTACAAG

TGCATGGTGGAAAACGTGGAAAAACTTTTCAATGCACGCAATGATCGACCCAGTGGG

>NvitOBP73(EMBL: HE578258)

CTAAAAGATGATGGTACACTGGATATTGAAGGTACAAGTATGATTGTAGGCCGAAGGCTA

AAGTATGCCGAGAGAAATGACGTAATCAAAGCAGACCAAGCTTGTTCTAATATTAAAGGA

GATAATGCATGTGATACCATTTTTAAGATTGTCGGATGCTCAATCAAAAACCTTGAGAGA

TATCGT

>NvitOBP74(EMBL: HE578259)

ATGATGGATATTGAAGGTTCTAGTATGATTGTAGGCAGAAGGCTAAAGGATGTCGAGAGA

AATGATGTAATCATAGCAGTCCAAGCTTGTTCTGATATTAAAGGAGATAATGCATGTGAT

ACCATTTTTAAGATTGTCGGATGCTCAATCAAAAACCTTAAGAGATATCGT

>NvitOBP75(EMBL: HE578260)

ATGAGAGTCTTGCTAGTCGTCGTGAGTGTATGCTTCGTTGGCAGCTATGCGGATTACGCG

GACGACATCAGAAAGCTCCAAGAAGAGACGAAGAGGATAGAGGAGTACAGGAGGCCCTGT

CTCAAGGAGGTCGGCCTCTACGCAGATCCGGCTAACGGAATCACCAGCCAGCCAGCGAGT

AGTCCCACGATAGGCCAGATCTTCTGTCTGTGGGCCTGTCTCTATCGCAAGAATGGATCC

ATAAGACCGGATGGCTCCGTGGACGAGGCAGCCGTCCGCTCTAAAAATCCCGAGCTCGAG

GGCCCGCTGGACGTGATAATAAGCAAGTGCGAGAATCAAGCTGGGGAAAATACTTGCAAG

CTCGCTGGCTGTCTAGCGAAAGCTCATTTTAATCTGTTAGAG

>NvitOBP76(EMBL: HE578261)

ATGATGCAAGGCTCCCTGTGCGCACTGGTCGTCTTGTCGCTGGTCTGCCTCGTCAGGGCT

GGGCCACCGGATTGGATCAGTGCTGAGATCCTGGAGATGGTGCAGTCTGATAAGGGCAGG

TGCATGGCAGAACACGGCACAACCGAGGCGTTGATCGACGACGTCAACAAGGGTAACCTG

CCGAACGACAAAGCTATCACTTGCTACATGTACTGCCTCTTCGAGGCTTTTAGTTTGGTC

GACGAAGAAGCGAACATCGAGGTTGAGATGTTGGTCGGCTTCCTGCCGGAGCACATGCAA

GCTGTGGCCAATGAACTGATCGACGTTTGCGCCAAGCTCGACGGTGCCGATGTTTGCGAT

AAGATGTACGTCATGGCCAAGTGTGTCATGGAGAAACGTCCCGATCTCTGGTTCATGCTG

>NvitOBP77(EMBL: HE578262)

ATGAAGATTGTTGTTCTGTGCCTCGTCGTTCTTAGCGCAGTGGCATGTGTTTCCGCCGGA

TACAGAGAATACCAAAATGCTTGTCTGGATGAAAATGGATTGACCAAAGAGGAGTTTTAT

GCGATGAAAAGAAATCAGGACCCTAGATCTGGCTGCGTCACGGCGTGTATCATGAAGAAA

AATGGATCTATGAAACACGGAATCATTGATGCGAGGGGAATCAAAAGAAGAATGAGAACA

CTTTTAGCTCCATTCATAAGCAAGGATAAACTTTACGAGAAAATCGATTACTGCGTTGAT

GAAGCCGAAAATCACGTAGGCGTATGCGAAAAAGCATACGTATTGCAAAAGTGCTTGCGG

ACTCCAAGAGCCAACAATGTTCAAGGCGAACGTCAAAAGATGATTGAC

>NvitOBP78(EMBL: HE578263)

ATGAAGACCATAGTTTTCACACTCTGCATGATGACTGTGGCAGTCACGTGTTCTCCCCGT

CCTGGAGGTCGTGGAGGTGGAAGTATGTTCAGCAGAGAGAGCGTAAAGAAATGCATGGCA

GAAATGGATATTAAAAGAGAGGATATTAAAACCCTGAAACAAAACAATGATCCAAAACTG

TCTTGCCTTAATGCATGTGCGATGACCAAAGAGGAGATAATGGACGAAGCCGGAAATATT

GATGCAGACAAGCTTATTAAAGCAACCTTAGAAATAGTCCAGAAGAAAAAACCCGATATA

AATGTTGAGGAATTAGAAACTGCCATGCTCTCTTGCATTGAAAAAGCCAAAGAAGTTGAA

GATAAGTGTATGAAGGCAAAGACCCTGGTTGTCTGCTCTCATGAATACTGGAAAGCTAAT

GTAAAGGGAAACCCGAGCAGCGCAGGTGGTGAAGAAGAA

>NvitOBP79(EMBL: HE578264)

ATGAAGCTGTTTTTTGTAACCCTCTGCGTATTATTTGCGGCTGTTTACGGAGCAACCAAG

TCGGATTCGAAATCGGAAAAGATATTCCATGAATGCTTAGAAGAAAACGATATTAAAGAA

AGTGATTTCAAAAACTTGGAAGGCAAGAAAGATCCGAAAATGCGTTGCCTCATGGCTTGC

ATTCTTGAAAAAGAAGGAGCTTTAAAAGATGGGGAAATTGATGGCGATGTAATCAAAAAA

GATATAATTGCTGAATTCACGGAGGTTGATGCACAAAAAATAAGCGATGCCATAGATACA

TGTGTCGATGGAGCAAACGATTTATCAGATATTTGCGAAAAAACTTCGTTTATTGGAGAA

TGTCTCAAGGTGGAATTGGACAAACTAGAAATGAATATGAAT

>NvitOBP80(EMBL: HE578265)

ATGGGAGGCTTCGTAACTGTTCTATATTTCTTAAGTATAATAATATGTGTTTACAGTCTA

AATTGGTCGGAAGCGAAAAAGCACGTTCAGGAGTGTTTGGACGAATACCAAATTACCAGA

GAAGATGTAGCTAAGTTGAAAAAAGAAGAATCGCCAGACTATAATTGTTACATAGCTTGT

ATAATGAAAAAGCGTGGATCTTTGGTAGATGGAAAAATCGACGAAGAAAAAATGTTGGAA

ATATTAAAACAACTACACGTATTGAATTCAGAACGAACCGAAGATAAATTTAGGATATGT

GCTACTGAAGCCAATAAACAGAGTAACGAATGTCTGGTTGCTGGAGATATGATAGGCTGT

TTGTATTTCAAATCTAAC

>NvitOBP81(EMBL: HE578266)

ATGAAAGTTATTGTATTGCTAGTTACTGTACTAACCATAACGATTCACGTTTCGTGTCAA

ACGGATGAAGAAGTACATAAAATAAAAGAAAAGTGTTTTGATCTGAGTGACATACCCGTA

GAAGACCGAGTAGTGTACAACCCTGAAAATCCAAAGCTCAAATGCTTTAATGCATGTACC

TACACTGGTGTAGGAATGATGAAGGATGGAAAAATAGTACCAGAAAAATACATCGAAAGA

CTGCAAGATTCTTTAAAAAATGAGAAGAAGTCGGACGTGGAAGCATTTATGAAACATATG

GAGGATTGTGCCGTTATGGCTAACAAACTAAGCGATGAGTGTGAAGTAGCCTATTCAATG

ATAAAATGTTTG

>NvitOBP82(EMBL: HE578267)

ATGAAGCGAGTTATGGCTTTAGTTGGCGCTTTTCTGCTGGTGAGTGCTGTGCAGTGCGAT

GATATGCCGTTTTGGAATGAGAAGGTGGAATGCGCTCAAAGCATGGGAATCAGTCCTGAC

CAAATGACGTCCATGTTGACGTCGAACGATGCACAAATGAATTGCGTACATGCCTGCGTG

TTAGAAAAAATAGGCGGAATGGTTGACGGCAAATTAAGTCTCGACAGCCTTATGGAATCT

CTAGAGAAACTTAAAGCTGAAGTCAAGGATTACGATGCAACTAAGGCTGGTATCCATCAG

TGCTTTGATCAAGCCAGCGGTGATAGATGCGAATCGGCCGGCAAATTCGCTATGTGCATG

CAGGAGCACATGCAAGGT

>NvitOBP83(EMBL: HE578268)

ATGAGATTAACATTACAACTGATAACTCTAGTATCTTTAGTAGCCATTTTCAAAACTACC

GAATCCAAAATGACCATGGACCAAATCAAAAATACTTTGAAACCATTCAAAAACTCATGT

ATTAAGAAAATTTCACCAGATGTAGCAATGGTGGAAGCAACCAAATCTGGACAATTCCCC

GAAGACGCGACCTTGATGTGCTTCTTAAAATGTGTTTTGTCAATGATGAAAGTGATGAAA

AATGGCGAAATCCTATTACCATCAATAATGCAACAAATAGATATTATGATGCCTGATGAA

TACGTCGAAACAATGAAAGAAATCTGTACCAATTGTTACGAGATGTCGCTGAAAGTTGAT

GATGCGTGTGAAAAGGCCTATGTTTTTGTCAAATGCTACTACAACACTAACAGCGAGCTG

TACTTCTTCCCA

>NvitOBP84(EMBL: HE578269)

ATGAGGAGGAGTATTTTGATAACGTCGATTCTGATCATCCTTATTTCGCAGTACAAACTA

GTCAAGTGTAAAAAAATGAATCTGGACGAATTGAGGGATATGTTGCGGCCCATGAGCAAA

TCGTGCAAAAGCAAAACGGGAGTTTCCGATGAAATGGTCGCGGCTACTCATCAAGGAATT

TTTCCCAGAGAAAAACCGCTCATGTGTTACTTCAAGTGTCTCTCGGTCATGTTGAAAGTC

ATGAACAAACAAGGTGAAATTAAGCCGAAAGATGTCGAACGACAAATCGACCTTTTGGTG

ATTCCCGAGCTTGCGCCGACTCTTAAAAAGATCGGAACCGACTGTTACAATAAAGTCGCA

CCAACTAATGACGCTTGTGCCTACGCTTTCGAAATTGTAATGTGCGGTTATCAGACGGAT

CCAAAGTATTACTTTTTACCA

>NvitOBP85(EMBL: HE578270)

ATGAGGTCCGTTCTGCTGATCTTTTGCCTGTCGTCCGTAGCGGTCCGCGTTTCCGCTCAC

GTTTCTCCCGTGGCCGACAGCTTCAAAGCGTGCTTGGCTGAAAGTGGAATGACTAGAGAC

GATTTTATCAAGGCCTTGCAGTCGAGCGACGACTCGAAAGCCCAGTGCATCGCGGCCTGT

ACGATGGAGAAGGAGAAATTCATGAGCGACGATAAGATAAACGTAGACGCGATAATAGCG

AAGATGGAGGACGTGTCGCAGGAGATTGGCAAAGTCCAGATAACGGATTTAGTGATGAAC

TGCGCAGCCGAAGCTAAGGACAAGAGCGGCAAGTGCGGCGTCGCTCACTCGGTCGTGAGG

TGCATACACGAGGAGTTGCGAAAGGAAGGCTGGATA

>NvitOBP86(EMBL: HE578271)

ATGAAAAGTTACTCGGTGATTCTGTTAGCCATTTGCTTTGCAGCGATCTATTCGAGTAGC

GCTCTTATATCGATAGAAGACAAGGCTGCATGTTTAAAAAAAAATGGATTAAACAATACT

GAAAAGTGGGATTTAACGGCACAATTCGATTATCGCTTGGAAAAGCCGTTTACGTGCTAT

GTTGCTTGCGTGATTAATGCGATCAAAAAGCCCGAGGAAACTGTTTATGGAAAGCTGAGC

GAAGTTATCGAACGCGGCCACGTAATTCCAGCTAGCCTCAAAAAAGACATGGAAAATCGT

TTGGATAGTTGCTATCGATATAATGGTGAAGGCGATGACTGTAAATTATTATACTGCGTC

AAGATACTCCAATCTCCATTAATCAAACTCTCAATTTACTCGCTTGAAGATATAGAACTT

>NvitOBP87(EMBL: HE578272)

ATGAAGTTTCTAATTTTTGTGATAAGTTTATTTACCGTCGTGGCAAGATCTCGGCAAAGC

CTTGCGGATATAGAAGCATGCGCTTCTCAATATGGAGTCGAAAACGTTACGAGGATTCCA

GATAACGATAGACCGTTCAAACAGAGAGATCCAGACTATGAATGCCTTCGTGCTTGTTTA

TGGAGGAAGCAAGGAATAATGAAAAACGGAAAATTCGATCTCGATAAGGCATTCAACTAT

TTCAAAAAGACTACAAGATTTCCTTTGACAGTCTTTAAAGAAAAACTAAGCGTCTGTGTT

GAGAAAGGAAATCAAGAAAAAAACGAGTGCGGTGTAACAAGAGTATACGTCGATTGCATG

AACGGTAGTCCGAAAGCACGGAAG

>NvitOBP88(EMBL: HE578273)

ATGAAGCTTCTTATTTTTGTGATAAGTTTTTTTATTGTTGCTGCGCATTCGCAGCCAAGA

AGTATGGACTGGAAAGGATGCATGGAGGAAATTGGAGTGAGCAAAGATGACGTTAAGTCA

ACTGAGTGGGGTGATCCTAAATCAAGATGCGTTCTTGCTTGCACGTTTAAAAAAGTGGGA

GTAATCAACGATGGCAAAGTGGTTTTTGACGTGGCATTTGATATAACCAAAGGAGAAGCC

CAAGATTCTTCACATGACAAATATATCGAAGAAAAAGTAAATAGCTGCATTGAAAAAGCA

CACCAAGAAACAAATGAATGTGATGTATCGTATGTCTTTATGGAATGCATGAAGACTAAT

AATAACACTGCAAAAATGGCAAATGGAACCATGTCAATA

>NvitOBP89(EMBL: HE578274)

GTAACCACCAACAATGATTTGTTTAAGATCAAAGATGCGAAAGTGGTTTCTAACGTGGCA

TTCGATATGGCTAAAGAAGATACACGAAGTTCAAGCGACAAAGATACACAAGAGAAAGTA

AACATGTGCATAGAAAAAGCACACCAAGAAGCAAACGAATGTGATGTAACATATGTATTT

TTGGACTGCCTTGCCGACGGATTAATAATGGCAAAGAAACAAGTTGTTAATATAAAA

>NvitOBP90(EMBL: HE578275)

ATGAAGAACCTTGCACTGCTTCTGCTGACTCTGTGCGTCGTCTCCTGCCTACTCATCAAT

GGAGCGCGAGCCGGAGTATCCCGAGAGCAGATGGAAAAAATGGCCAACGGCTTTCGAAAC

ACCTGCGTCGGAAAGACCGGAGCCGATATGTCGCTCGTCGAGGGAATAAGGGTCGGCAAT

TTCGTCGAAGATCCAACCTCCATGTGCTACACCAAGTGCATCATGGGCCTCATGAAAACG

TTCACGAAGCAAGGCAACATCGACGTCGAGATGCTGGTCAAACAGATCAATGTCATGGCC

TCGCCGGACATAGCCGGGAGCATGGTCACCAACGCGAGGAAATGTCACGCCGAAACGTCA

GCCAGTGATCCCTGCGAACTCGCCTGGCTCTTCACCAAGTGCATCTACGCTGCTGATCCA

GCGGTCTACTTCTTCCCG
