# Supplementary material for: Unique Features of Odorant-Binding Proteins of the Parasitoid Wasp Nasonia vitripennis Revealed by Genome Annotation and Comparative Analyses
Source: PLoS One. 2012 Aug 27;7(8):e43034. doi: 10.1371/journal.pone.0043034 (PMC3428353; doi:10.1371/journal.pone.0043034)
Supplement: Table S1 — Accession numbers of protein sequences cited in the manuscript. (PDF) [file pone.0043034.s005.pdf]

| Gene | Accession Number |
|---|---|
| NvitOBP1 | EMBL: HE578186 |
| NvitOBP2 | EMBL: HE578187 |
| NvitOBP3 | EMBL: HE578188 |
| NvitOBP4 | EMBL: HE578189 |
| NvitOBP5 | EMBL: HE578190 |
| NvitOBP6 | EMBL: HE578191 |
| NvitOBP7 | EMBL: HE578192 |
| NvitOBP8 | EMBL: HE578193 |
| NvitOBP9 | EMBL: HE578194 |
| NvitOBP10 | EMBL: HE578195 |
| NvitOBP11 | EMBL: HE578196 |
| NvitOBP12 | EMBL: HE578197 |
| NvitOBP13 | EMBL: HE578198 |
| NvitOBP14 | EMBL: HE578199 |
| NvitOBP15 | EMBL: HE578200 |
| NvitOBP16 | EMBL: HE578201 |
| NvitOBP17 | EMBL: HE578202 |
| NvitOBP18 | EMBL: HE578203 |
| NvitOBP19 | EMBL: HE578204 |
| NvitOBP20 | EMBL: HE578205 |
| NvitOBP21 | EMBL: HE578206 |
| NvitOBP22 | EMBL: HE578207 |
| NvitOBP23 | EMBL: HE578208 |
| NvitOBP24 | EMBL: HE578209 |
| NvitOBP25 | EMBL: HE578210 |
| NvitOBP26 | EMBL: HE578211 |
| NvitOBP27 | EMBL: HE578212 |
| NvitOBP28 | EMBL: HE578213 |
| NvitOBP29 | EMBL: HE578214 |
| NvitOBP30 | EMBL: HE578215 |
| NvitOBP31 | EMBL: HE578216 |
| NvitOBP32 | EMBL: HE578217 |
| NvitOBP33 | EMBL: HE578218 |
| NvitOBP34 | EMBL: HE578219 |
| NvitOBP35 | EMBL: HE578220 |
| NvitOBP36 | EMBL: HE578221 |
| NvitOBP37 | EMBL: HE578222 |
| NvitOBP38 | EMBL: HE578223 |
| NvitOBP39 | EMBL: HE578224 |
| NvitOBP40 | EMBL: HE578225 |
| NvitOBP41 | EMBL: HE578226 |
| NvitOBP42 | EMBL: HE578227 |
| NvitOBP43 | EMBL: HE578228 |
| NvitOBP44 | EMBL: HE578229 |
| NvitOBP45 | EMBL: HE578230 |
| NvitOBP46 | EMBL: HE578231 |
| NvitOBP47 | EMBL: HE578232 |
| NvitOBP48 | EMBL: HE578233 |
| NvitOBP49 | EMBL: HE578234 |

| Gene | Accession Number |
|---|---|
| AmelOBP1 | GenBank: AAL60419 |
| AmelOBP2 | GenBank: AAL60418 |
| AmelOBP3 | GenBank: ABD92639 |
| AmelOBP4 | GenBank: AAL60420 |
| AmelOBP5 | GenBank: AAL60422 |
| AmelOBP6 | GenBank: AAL60421 |
| AmelOBP7 | GenBank: ABD92640 |
| AmelOBP8 | GenBank: AAK01304 |
| AmelOBP9 | GenBank: ABD92641 |
| AmelOBP10 | GenBank: ABD92642 |
| AmelOBP11 | GenBank: ABD92643 |
| AmelOBP12 | GenBank: ABD92644 |
| AmelOBP13 | GenBank: ABD92645 |
| AmelOBP14 | GenBank: ABD92646 |
| AmelOBP15 | GenBank: ABD92647 |
| AmelOBP16 | GenBank: ABD92648 |
| AmelOBP17 | GenBank: ABD92649 |
| AmelOBP18 | GenBank: ABD92650 |
| AmelOBP19 | GenBank: ABD92651 |
| AmelOBP20 | GenBank: ABD92652 |
| AmelOBP21 | GenBank: ABD92653 |

| | |
|---|---|
| NvitOBP50 | EMBL: HE578235 |
| NvitOBP51 | EMBL: HE578236 |
| NvitOBP52 | EMBL: HE578237 |
| NvitOBP53 | EMBL: HE578238 |
| NvitOBP54 | EMBL: HE578239 |
| NvitOBP55 | EMBL: HE578240 |
| NvitOBP56 | EMBL: HE578241 |
| NvitOBP57 | EMBL: HE578242 |
| NvitOBP58 | EMBL: HE578243 |
| NvitOBP59 | EMBL: HE578244 |
| NvitOBP60 | EMBL: HE578245 |
| NvitOBP61 | EMBL: HE578246 |
| NvitOBP62 | EMBL: HE578247 |
| NvitOBP63 | EMBL: HE578248 |
| NvitOBP64 | EMBL: HE578249 |
| NvitOBP65 | EMBL: HE578250 |
| NvitOBP66 | EMBL: HE578251 |
| NvitOBP67 | EMBL: HE578252 |
| NvitOBP68 | EMBL: HE578253 |
| NvitOBP69 | EMBL: HE578254 |
| NvitOBP70 | EMBL: HE578255 |
| NvitOBP71 | EMBL: HE578256 |
| NvitOBP72 | EMBL: HE578257 |
| NvitOBP73 | EMBL: HE578258 |
| NvitOBP74 | EMBL: HE578259 |
| NvitOBP75 | EMBL: HE578260 |
| NvitOBP76 | EMBL: HE578261 |
| NvitOBP77 | EMBL: HE578262 |
| NvitOBP78 | EMBL: HE578263 |
| NvitOBP79 | EMBL: HE578264 |
| NvitOBP80 | EMBL: HE578265 |
| NvitOBP81 | EMBL: HE578266 |
| NvitOBP82 | EMBL: HE578267 |
| NvitOBP83 | EMBL: HE578268 |
| NvitOBP84 | EMBL: HE578269 |
| NvitOBP85 | EMBL: HE578270 |
| NvitOBP86 | EMBL: HE578271 |
| NvitOBP87 | EMBL: HE578272 |
| NvitOBP88 | EMBL: HE578273 |
| NvitOBP89 | EMBL: HE578274 |
| NvitOBP90 | EMBL: HE578275 |
| NvitOBP91 | EMBL: HE578276 |
| NvitOBP92 | EMBL: HE578277 |
| NvitOBP93 | EMBL: HE578278 |

| **Gene** | **Accession Number** | **Gene** | **Accession Number** |
|---|---|---|---|
| AcerASP1 | GenBank: ABD97847 | DmelOBP8a | GenBank: NP_727322 |
| AcerASP2 | GenBank: ABD97844 | DmelOBP18a | GenBank: NP_573350 |
| AcerASP4 | GenBank: AAR83081 | DmelOBP19a | GenBank: NP_728338 |
| MmedOBP1 | GenBank: ABM05968 | DmelOBP19b | GenBank: NP_608391 |

| | |
|---|---|
| MmedOBP2 | GenBank: ABM05969 |
| MmedOBP3 | GenBank: ABM05970 |
| MmedOBP4 | GenBank: ABM05971 |
| MmedOBP5 | GenBank: ABM05972 |
| SinvOBP11 | GenBank: ADX94407 |
| SinvOBP4 | GenBank: ADX94400 |
| SinvOBP5 | GenBank: ADX94401 |
| SinvOBP6 | GenBank: ADX94402 |
| SinvOBP7 | GenBank: ADX94403 |
| SinvOBP8 | GenBank: ADX94404 |

| **Gene** | **Accession Number** |
|---|---|
| AgamOBP1 | GenBank: AAO12081 |

| | |
|---|---|
| DmelOBP19c | GenBank: NP_608392 |
| DmelOBP19d | GenBank: ACY93964 |
| DmelOBP22a | GenBank: NP_722746 |
| DmelPBPRP5 | GenBank: NP_523505 |
| DmelOBP44a | GenBank: NP_610358 |
| DmelOBP46a | GenBank: NP_610574 |
| DmelOBP47a | GenBank: NP_995810 |
| DmelOBP47b | GenBank: NP_610669 |
| DmelOBP49a | GenBank: NP_610812 |
| DmelOBP50a | GenBank: NP_725385 |
| DmelOBP50b | GenBank: NP_725386 |
| DmelOBP50c | GenBank: NP_725387 |
| DmelOBP50d | GenBank: NP_725388 |
| DmelOBP50e | GenBank: NP_610959 |
| DmelOBP51a | GenBank: NP_725436 |
| DmelOBP56a | GenBank: NP_611442 |
| DmelOBP56b | GenBank: NP_611443 |
| DmelOBP56c | GenBank: NP_725925 |
| DmelOBP56d | GenBank: NP_611444 |
| DmelOBP56e | GenBank: NP_611445 |
| DmelOBP56f | GenBank: NP_725926 |
| DmelOBP56g | GenBank: NP_611447 |
| DmelOBP56h | GenBank: NP_611448 |
| DmelOBP56i | GenBank: NP_725929 |
| DmelOBP57a | GenBank: NP_725966 |
| DmelOBP57b | GenBank: NP_725965 |
| DmelOBP57c | GenBank: NP_611481 |
| DmelOBP57d | GenBank: NP_725973 |
| DmelOBP57e | GenBank: NP_611488 |
| DmelOBP58b | GenBank: NP_611709 |
| DmelOBP58c | GenBank: NP_611710 |
| DmelOBP58d | GenBank: NP_611711 |
| DmelOBP59a | GenBank: NP_788429 |
| DmelPBPRP1 | GenBank: NP_524039 |
| DmelLUSH | GenBank: NP_524162 |
| DmelPBPRP3 | GenBank: NP_524241 |
| DmelOBP83b | GenBank: NP_524242 |
| DmelOBP83cd | GenBank: AAF51919 |
| DmelOBP83ef | GenBank: AAF51918 |
| DmelOBP83g | GenBank: NP_731043 |
| DmelPBPRP4 | GenBank: NP_476990 |
| DmelOBP85a | GenBank: NP_649802 |
| DmelOBP93a | GenBank: NP_996254 |
| DmelOBP99a | GenBank: NP_651707 |
| DmelOBP99b | GenBank: NP_651713 |
| DmelOBP99c | GenBank: NP_651711 |
| DmelOBP99d | GenBank: NP_651712 |

| **Gene** | **Accession Number** |
|---|---|
| BmorOBP1(GOBP1) | GenBank: NP_001037496 |

| | |
|---|---|
| AgamOBP2 | GenBank: AAO12079 |
| AgamOBP3 | GenBank: AAO12105 |
| AgamOBP4 | GenBank: AAO12091 |
| AgamOBP5 | GenBank: AAO12089 |
| AgamOBP6 | GenBank: AAO12085 |
| AgamOBP7 | GenBank: AAO12102 |
| AgamOBP8 | GenBank: AAO12104 |
| AgamOBP9 | GenBank: AAO12100 |
| AgamOBP10 | GenBank: AAO12101 |
| AgamOBP11 | GenBank: AAO12103 |
| AgamOBP12 | GenBank: AAO12076 |
| AgamOBP13 | GenBank: AAO12078 |
| AgamOBP14 | GenBank: AAO12077 |
| AgamOBP15 | GenBank: AAO12080 |
| AgamOBP16 | GenBank: AAO12082 |
| AgamOBP17 | GenBank: AAO12083 |
| AgamOBP18 | GenBank: AAO12084 |
| AgamOBP19 | GenBank: AAO12086 |
| AgamOBP20 | GenBank: AAO12087 |
| AgamOBP21 | GenBank: AAO12088 |
| AgamOBP22 | GenBank: AAO12090 |
| AgamOBP23 | GenBank: AAO12093 |
| AgamOBP24 | GenBank: AAO12094 |
| AgamOBP25 | GenBank: AAO12095 |
| AgamOBP26 | GenBank: AAO12096 |
| AgamOBP27 | GenBank: AAO12097 |
| AgamOBP28 | GenBank: AAO12098 |
| AgamOBP29 | GenBank: AAO12099 |
| AgamOBP30 | GenBank: AAO12073 |
| AgamOBP31 | GenBank: AAO12075 |
| AgamOBP32 | GenBank: AAO12070 |
| AgamOBP33 | GenBank: AAO12069 |
| AgamOBP34 | GenBank: AAO12068 |
| AgamOBP35 | GenBank: AAO12067 |
| AgamOBP36 | GenBank: AAO12066 |
| AgamOBP37 | GenBank: AAO12065 |
| AgamOBP38 | GenBank: AAO12064 |
| AgamOBP39 | GenBank: AAO12072 |
| AgamOBP40 | GenBank: AAO12071 |
| AgamOBP41 | GenBank: AAO12063 |
| AgamOBP42 | GenBank: AAO12062 |
| AgamOBP43 | GenBank: AAO12061 |
| AgamOBP44 | GenBank: AAO12092 |
| AgamOBP45 | GenBank: AAO12074 |
| AgamOBP46 | GenBank: AAQ16279 |
| AgamOBP47 | GenBank: AAQ16280 |
| AgamOBP48 | GenBank: AAQ16281 |
| AgamOBP49 | GenBank: AAQ16282 |
| AgamOBP50 | GenBank: AAQ16283 |
| AgamOBP51 | GenBank: AAQ16284 |

| | |
|---|---|
| BmorOBP2(GOBP2) | GenBank: NP_001037498 |
| BmorOBP3(PBP1) | GenBank: CAA64443 |
| BmorOBP4(PBP2) | GenBank: CAL47308 |
| BmorOBP5 | GenBank: Unpublished |
| BmorOBP6(PBP3) | GenBank: CAL47309 |
| BmorOBP7 | GenBank: Unpublished |
| BmorOBP8 | GenBank: BAI44701 |
| BmorOBP9 | GenBank: Unpublished |
| BmorOBP10 | GenBank: Unpublished |
| BmorOBP11 | GenBank: BAH36759 |
| BmorOBP12 | GenBank: NP_001153664 |
| BmorOBP13 | Unpublished |
| BmorOBP14 | GenBank: CAS90125 |
| BmorOBP15 | GenBank: Unpublished |
| BmorOBP16 | Unpublished |
| BmorOBP17 | Unpublished |
| BmorOBP18 | Unpublished |
| BmorOBP19 | Unpublished |
| BmorOBP20(ABPX) | GenBank: CAA64446 |
| BmorOBP21 | Unpublished |
| BmorOBP22 | Unpublished |
| BmorOBP23 | GenBank: NP_001153665 |
| BmorOBP25 | Unpublished |
| BmorOBP26 | GenBank: NP_001159622 |
| BmorOBP27 | Unpublished |
| BmorOBP28 | Unpublished |
| BmorOBP29 | Unpublished |
| BmorOBP30 | Unpublished |
| BmorOBP31 | Unpublished |
| BmorOBP32 | Unpublished |
| BmorOBP33 | Unpublished |
| BmorOBP34 | Unpublished |
| BmorOBP35 | Unpublished |
| BmorOBP36 | GenBank: CAS90126 |
| BmorOBP37 | GenBank: CAS90127 |
| BmorOBP38 | GenBank: CAS90131 |
| BmorOBP39 | Unpublished |
| BmorOBP40 | Unpublished |
| BmorOBP41 | Unpublished |
| BmorOBP42 | GenBank: NP_001159621 |
| BmorOBP43 | GenBank: NP_001159621 |
| BmorOBP44 | Unpublished |

| | |
|---|---|
| AgamOBP52 | GenBank: AAQ16278 |
| AgamOBP53 | GenBank: AAQ16285 |
| AgamOBP54 | GenBank: AAQ16286 |
| AgamOBP55 | GenBank: AAQ16287 |
| AgamOBP56 | GenBank: AAQ16288 |
| AgamOBP57 | GenBank: AAQ16289 |
| AgamOBPjj11 | GenBank: CAG26914 |
| AgamOBP59 | GenBank: EAL38693 |
| AgamOBPjj9 | GenBank: CAG26912 |
| AgamOBPjj4 | GenBank: CAF01999 |
| AgamOBP62 | GenBank: XP_001237096 |
| AgamOBPjj5c | GenBank: CAF02002 |
| AgamOBPjj16 | GenBank: CAG26919 |
| AgamOBP65 | GenBank: XP_564945 |
| AgamOBP66 | GenBank: XP_001688146 |

| **Gene** | **Accession Number** |
|---|---|
| TcasOBP1 | GenBank: XP_975684 |
| TcasOBP2 | GenBank: XP_001810456 |
| TcasOBP2a | GenBank: XP_973884 |
| TcasOBP2b | GenBank: EFA05676 |
| TcasOBP3 | GenBank: EFA05675 |
| TcasOBP4 | GenBank: EFA05742 |
| TcasOBP5 | GenBank: EFA05677 |
| TcasOBP6 | GenBank: EFA04594 |
| TcasOBP7 | GenBank: EFA04593 |
| TcasOBP8 | GenBank: EFA04687 |
| TcasOBP9 | GenBank: EFA10713 |
| TcasASP | GenBank: XP_001813655 |
| TcasASP1 | GenBank: XP_001808838 |
| TcasASP2 | GenBank: XP_968742 |
| TcasASP3 | GenBank: XP_968815 |
| TcasASP4 | GenBank: XP_001808902 |
| TcasPBP | GenBank: XP_968365 |
| TcasOBP12 | GenBank: EFA02857 |
| TcasOBP13 | GenBank: EFA02858 |
| TcasOBP14 | GenBank: EFA02914 |
| TcasOBP15 | GenBank: EFA12066 |
| TcasOBP16 | GenBank: EFA02853 |
| TcasOBP17 | GenBank: EFA02861 |
| TcasOBP18 | GenBank: EFA02860 |
| TcasOBP19 | GenBank: EFA02960 |
| TcasOBP20 | GenBank: EFA05793 |
| TcasOBP21 | GenBank: EFA09215 |
| TcasOBP23 | GenBank: EFA10803 |
| TcasOBP24 | GenBank: EFA04576 |
| TcasOBP25 | GenBank: EFA04747 |
| TcasOBP26 | GenBank: EFA04746 |

| **Gene** | **Accession Number** |
|---|---|
| ApisOBP1 | GenBank: CAR85628 |
| ApisOBP2 | GenBank: CAR85629 |
| ApisOBP3 | GenBank: CAR85630 |
| ApisOBP4 | GenBank: CAR85631 |
| ApisOBP5 | GenBank: CAR85632 |
| ApisOBP6 | GenBank: CAR85633 |
| ApisOBP7 | GenBank: CAR85634 |
| ApisOBP8 | GenBank: CAR85635 |
| ApisOBP9 | GenBank: CAR85636 |
| ApisOBP10 | GenBank: CAR85637 |
| ApisOBP11 | GenBank: CAX63068 |
| ApisOBP12 | GenBank: CAX63069 |
| ApisOBP13 | GenBank: CAX63070 |
| PhumOBP1 | GenBank: XP_002430486 |
